# Identification and analysis of phosphorylation status of proteins in dormant terminal buds of poplar

**DOI:** 10.1186/1471-2229-11-158

**Published:** 2011-11-11

**Authors:** Chang-Cai Liu, Chang-Fu Liu, Hong-Xia Wang, Zhi-Ying Shen, Chuan-Ping Yang, Zhi-Gang Wei

**Affiliations:** 1 State Key Laboratory of Forest Genetics and Tree Breeding (Northeast Forestry University), 26 Hexing Road, Harbin 150040, China; 2 Laboratory for Chemical Defence and Microscale Analysis, P.O. Box 3, Zhijiang 443200, China; 3 Shenyang Agricultural University, Dongling Road 120, Shenyang, Liaoning 110866, China; 4 Institute of Basic Medical Sciences, National Center for Biomedical Analysis, 27 Taiping Road, Beijing 100850, China; 5 Daqing Branch, Harbin Medical University, Daqing 163319, China

## Abstract

**Background:**

Although there has been considerable progress made towards understanding the molecular mechanisms of bud dormancy, the roles of protein phosphorylation in the process of dormancy regulation in woody plants remain unclear.

**Results:**

We used mass spectrometry combined with TiO_2 _phosphopeptide-enrichment strategies to investigate the phosphoproteome of dormant terminal buds (DTBs) in poplar (*Populus simonii × P. nigra*). There were 161 unique phosphorylated sites in 161 phosphopeptides from 151 proteins; 141 proteins have orthologs in *Arabidopsis*, and 10 proteins are unique to poplar. Only 34 sites in proteins in poplar did not match well with the equivalent phosphorylation sites of their orthologs in *Arabidopsis*, indicating that regulatory mechanisms are well conserved between poplar and *Arabidopsis*. Further functional classifications showed that most of these phosphoproteins were involved in binding and catalytic activity. Extraction of the phosphorylation motif using Motif-X indicated that proline-directed kinases are a major kinase group involved in protein phosphorylation in dormant poplar tissues.

**Conclusions:**

This study provides evidence about the significance of protein phosphorylation during dormancy, and will be useful for similar studies on other woody plants.

## Background

Dormancy is a key feature of perennial plants. During dormancy the meristem becomes insensitive to growth-promoting signals for a period of time, before it is released and growth resumes [1,2]. Bud dormancy is a critical developmental process that allows perennial plants to survive extreme seasonal variations in climate. The regulation of dormancy is a complex process that is necessary for plant survival, development, and architecture [3,4]. A thorough understanding of regulation mechanisms controlling dormancy in woody perennials would have a variety of applications for genetic improvement of woody trees [3,5,6]. Considerable progress has been made in understanding the molecular mechanisms and regulatory pathways involved in bud dormancy [2]. However, until recently such studies focused on regulation at the levels of transcription, post-transcription, and translation [1,7-12]. Despite the importance of dormancy regulation for perennial behavior [3], the roles of post-translational modifications, especially protein phosphorylation, remain poorly understood.

The identification of phosphorylation sites within a certain protein cannot provide a comprehensive view of the regulatory role of protein phosphorylation [13-17]. Instead, the simultaneous identification of the phosphorylation status of numerous proteins at a certain developmental stage is required to decode regulatory mechanisms. Large-scale mapping of phosphorylations that occur in response to diverse environmental signals has become an indispensable method for unraveling plant regulatory networks [17-22]. In recent years, advances in mass spectrometry (MS)-based protein analysis technologies, combined with phosphopeptide enrichment methods, paved the way for large-scale mapping of phosphorylation sites *in vivo *[13,18,23]. Specifically, the titanium dioxide (TiO_2_) microcolumn is an effective method to selectively enrich phosphopeptides [17,24-28]. There have been several studies on plant phosphoproteomes. These studies have provided large datasets that allow new insights into phosphorylation events; however, they have been carried out only on herbaceous plants, such as *Arabidopsis *[22,29-40], oilseed rape [41], rice [42], barley [43], and maize [44]. To date, there have been no reports on the phosphoproteomes of woody plant species, except for the identification of eight phosphorylated poplar P-proteins [45].

Numerous cellular signaling pathways are based on the sequential phosphorylation of an array of proteins [15,33,46]. Therefore, the analysis of signaling pathways in plants has often focused on protein kinases. Kinases show catalytic preferences for specific phosphorylation motifs with certain amino acid context sequences [33,47,48]. Therefore, identification of *in vivo *phosphorylation sites can provide important information about the activity of protein kinases in their cellular context.

To better understand the regulation mechanism of phosphoproteins and cellular signaling networks during dormancy, we investigated the phosphoproteome of dormant terminal buds (DTBs) of hybrid poplar (*Populus simonii × P. nigra*) using a MS method combined with a TiO_2 _phosphopeptide enrichment strategy. We identified 161 phosphorylation sites in 161 phosphopeptides from 151 proteins, most of which are associated with binding and catalytic activity. The information gained from this study provides a wealth of resources and novel insights to decode the complicated mechanisms of phosphorylation modifications in poplar. As far as we know, this is the first phosphoproteomic analysis of woody plants.

## Results

### Identification and characterization of the phosphoproteome of DTBs

Total proteins were isolated from DTBs of poplar, and then digested with trypsin in solution. The resulting tryptic peptides were subjected to nanoUPLC-ESI-MS/MS to identify phosphorylation modifications after TiO_2 _enrichment. In total, 161 unique phosphorylation sites were identified in 161 phosphopeptides from 151 proteins (Table 1, Additional file 1, Additional file 2 and Additional file 3).

**Table 1:** Characterization of identified phosphopeptides, phosphoproteins, and phosphosites

Items	Number
Phosphopeptides^1^	161
Phosphoproteins	151
Phosphorylation sites	161
Phosphorylated residues (Ser: Thr: Tyr)	131: 28: 2
	(81.3%) (17.4%) (1.2%)

^1^Number of phosphopeptides counted according to unique sequences containing oxidized methionine or acetylated/phosphorylated residues.

**Figure 1:**
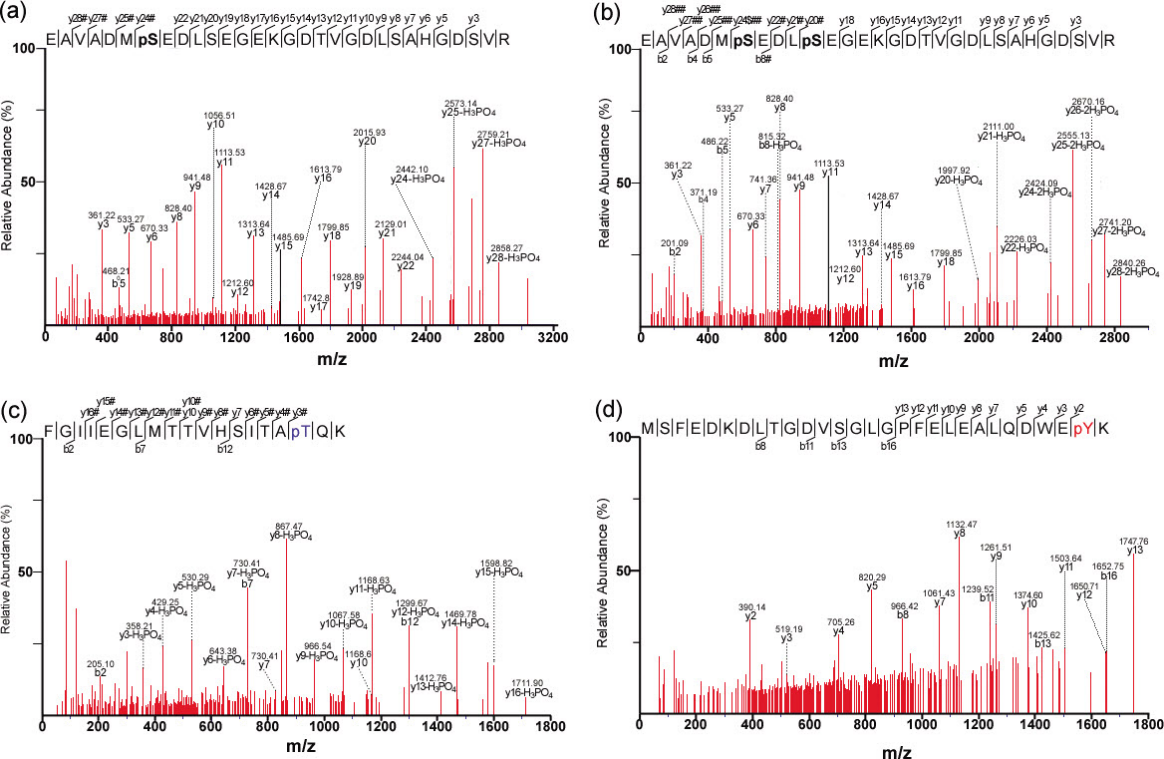
**MS/MS spectra of poplar phosphopeptides with single or double phosphorylations**. ESI-QUAD-TOF tandem MS spectra of doubly charged parent molecular ions with 780.30 m/z. b-type and y-type ions, including H_3_PO_4 _neutral loss ions (indicated as -H_3_PO_4 _and # in spectra), were labeled to determine peptide sequences and to localize phosphorylation sites. Asterisks denote phosphorylated serine, threonine, or tyrosine residues. (a) Phosphopeptide spectrum of EAVADMS*EDLSEGEKGDTVGDLSAHGDSVR with a single pSer, corresponding to glycosyltransferase (578888). (b) Phosphopeptide spectrum of EAVADMS*EDLS*EGEKGDTVGDLSAHGDSVR containing two phosphorylated Ser residues, corresponding to glycosyltransferase (578888). (c) Phosphopeptide spectrum of FGIIEGLMTTVHSITAT*QK with a single pThr, corresponding to glyceraldehyde 3-phosphate dehydrogenase (728998). (d) Phosphopeptide spectrum of MSFEDKDLTGDVSGLGPFELEALQDWEY*K with a single pTyr, corresponding to cytochrome b5 domain-containing proteins (662371 and 666994).

Among these phosphorylation sites, 81.3% (131) of phosphorylation events occurred on Ser and 17.4% (28) on Thr (Table 1). This finding is consistent with previously reported phosphorylation patterns: 85% pSer and 10.6% pThr [22] and 88% pSer and 11% pThr [33] in *Arabidopsis*; and 86% pSer and 12.7% pThr in *M. truncatula *[49]. Only 1.2% (2) of the phosphorylation events of these phosphopeptides occurred on Tyr residue. This is lower than the pTyr values reported for *Arabidopsis *(4.2%) and rice (2.9%) [22,50], but comparable to that reported for *Medicago truncatula *(1.3%) [49]. The results of these studies indicate that Tyr phosphorylation in plants is more abundant than once thought [51]. The spectra representing all phosphopeptides and the original detailed data are shown in Additional file 4. As examples, the spectra of phosphopeptides with single pSer, pThr, and pTyr are shown in Figure 1a, c, and 1d, respectively. The spectrum of a phosphopeptide containing two phosphorylated Ser residues is shown in Figure 1b.

The majority (93.8%) of the 161 phosphopeptides were phosphorylated at a single residue. This value is higher than that reported for *Arabidopsis *(80.9%) [22] and *M. truncatula *(66.4%) [49]. Only 6.2% of the phosphopeptides from poplar contained two phosphorylated residues, and none were phosphorylated at multiple sites. In *Arabidopsis *and *M. truncatula*, 19.1 and 27.1% of phosphopeptides, respectively, were doubly phosphorylated [22,49] (Additional file 5). This may be a result of different enrichment strategies that show selective or preferred affinity for single or multiple phosphopeptides [52,53].

In a recent phosphorylation mapping study in *Arabidopsis*, the phosphorylation sites were concentrated outside conserved domains [22,30]. To evaluate whether this pattern also occurred among poplar phosphopeptides, we conducted Pfam searches [54] to obtain domain information for the 151 phosphoproteins. We acquired domain information of 134 phosphoproteins (Additional file 1). These data showed that 81.9% of the phosphorylation sites were located outside of conserved domains (Additional file 6), consistent with previous results [22,30]. Protein phosphorylation often leads to structural changes in proteins, and such changes can directly modulate protein activity and reflect changes in interaction partners or subcellular localization [14]. Thus, phosphorylations outside conserved domains can be expected to alter protein conformation and functions.

### Conservation of phosphoproteins and phosphosites between poplar and *Arabidopsis*

We compared phosphorylation patterns of orthologous proteins between poplar and *Arabidopsis *to analyze conservation between their phosphoproteomes. Additional file 7 shows orthologous proteins in poplar and *Arabidopsis*. Phosphorylation sites in poplar that were absent from their equivalent sites in proteins from other plant species were considered to be novel phosphorylation sites (Additional file 2).

**Figure 2:**
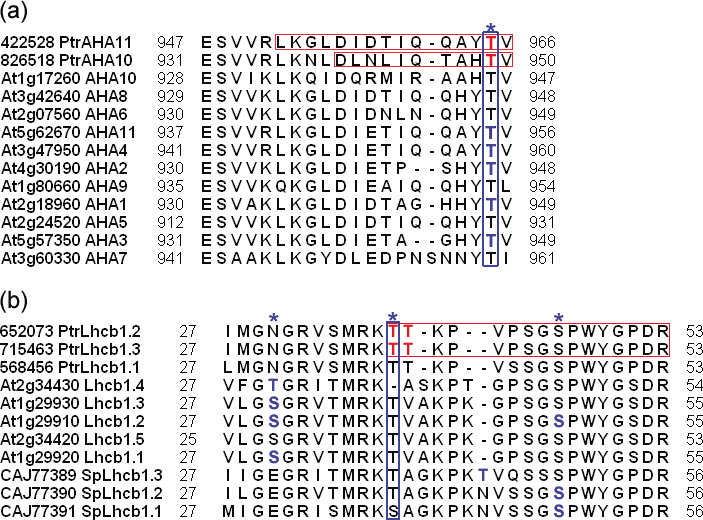
**Conservation of phosphorylation sites between poplar proteins and homologs in other plants**. Sequence alignments were conducted to determine conservation of phosphorylation sites among homologs. Gaps were introduced to ensure maximum identity. Fine red boxes represent phosphopeptides identified in this study. Phosphorylation sites identified in our study are shown in red bold font. Previously identified phosphorylation sites in *Arabidopsis *are indicated blue bold font. Well-conserved phosphorylation sites are shown within blue box in bold. Phosphorylation site is marked with an asterisk. (a) Phosphorylation sites conserved across plant plasma membrane H+-ATPases (AHA) orthologs. (b) Phosphorylation sites conserved across plant chlorophyll-a/b-binding protein 1 (Lhcb1) orthologs.

We found only 10 phosphoproteins that were unique to poplar, and the rest had ortholog(s) in *Arabidopsis*. Among these ortholog(s), more than 75% (110) were phosphoproteins, and almost half of them were phosphorylated at equivalent site(s) or neighboring site(s) in poplar and *Arabidopsis *(Table 2; Table 3). Among the identified phosphosites, 127 (84.1%) were conserved across the two species. The proteins containing these sites were involved in various physiological processes (see Additional file 8). Of the 127 conserved sites, only 62 were phosphorylated in the *Arabidopsis *ortholog(s), and the remaining 65 were novel phosphorylation sites in poplar (Additional files 8 and 9). Note that the residues at the equivalent sites of ortholog(s) are potential phosphorylation sites, as shown in Additional file 8. For example, two different poplar plasma membrane H+-ATPase isoforms (PtrAHA10, 826518 and PtrAHA11, 422528) and their *Arabidopsis *homologs (At1g17260 and At5g62670) were phosphorylated at their well-conserved C-terminal domain (Figure 2a). In *Populus trichocarpa*, the Lhcb1 protein exists as three distinct isoforms; Lhcb1.1 (568456), Lhcb1.2 (652073) and Lhcb1.3 (715463). In the present study, we identified two previously unknown phosphorylation sites at the N-terminus; Thr38, which is well conserved across the Lhcb1 isoforms of several plants, and Thr39, which is not conserved across Lhcb1 isoforms of other plants, but is present as a non-phosphorylated residue in the Lhcb1 isoforms of *Arabidopsis *and spinach (Figure 2b).

**Table 2:** Conservation of phosphosites and phosphoproteins between poplar and *Arabidopsis*

Phosphoproteins	Number
1) Proteins unique to poplar	10
2) Proteins with ortholog(s) in *Arabidopsis*	141
3) Proteins whose ortholog(s) are not phosphorylated	31
4) Proteins whose ortholog(s) are phosphorylated	110
5) Equivalent site(s) are phosphorylated in ortholog(s)	62
6) Other site(s) are phosphorylated in ortholog(s)	48

**Table 3:** Similarities of phosphoproteins/phosphosites conserved between poplar and *Arabidopsis*

Similarity with closest homologs in *Arabidopsis*	Number of phosphoproteins	Number of phosphosites	Conservation of phosphosites		Phosphosites in *Arabidopsis *counterparts	
			
			Unconserved	Conserved	Undescribed	Described
70-100%	124	132	18	114	53	61
50-70%	17	19	6	13	12	1
<50%	8	7	7	0	7	0
No similarity	2	3	3	0	3	0
Total	151	161	34	127	75	62

Recently, overlaps among *Medicago*, rice, and *Arabidopsis *phosphoproteomes suggested that the phosphoproteomes are similarly conserved among various herbaceous plant species, and that overlaps are not specifically dependent on experimental conditions [50]. In this work, we observed overlaps between the poplar and *Arabidopsis *phosphoproteomes, providing additional evidence that phosphoproteomes overlap across plant kingdoms.

### Unique phosphorylation sites of poplar proteins, compared with orthologs in other plants

**Figure 3:**
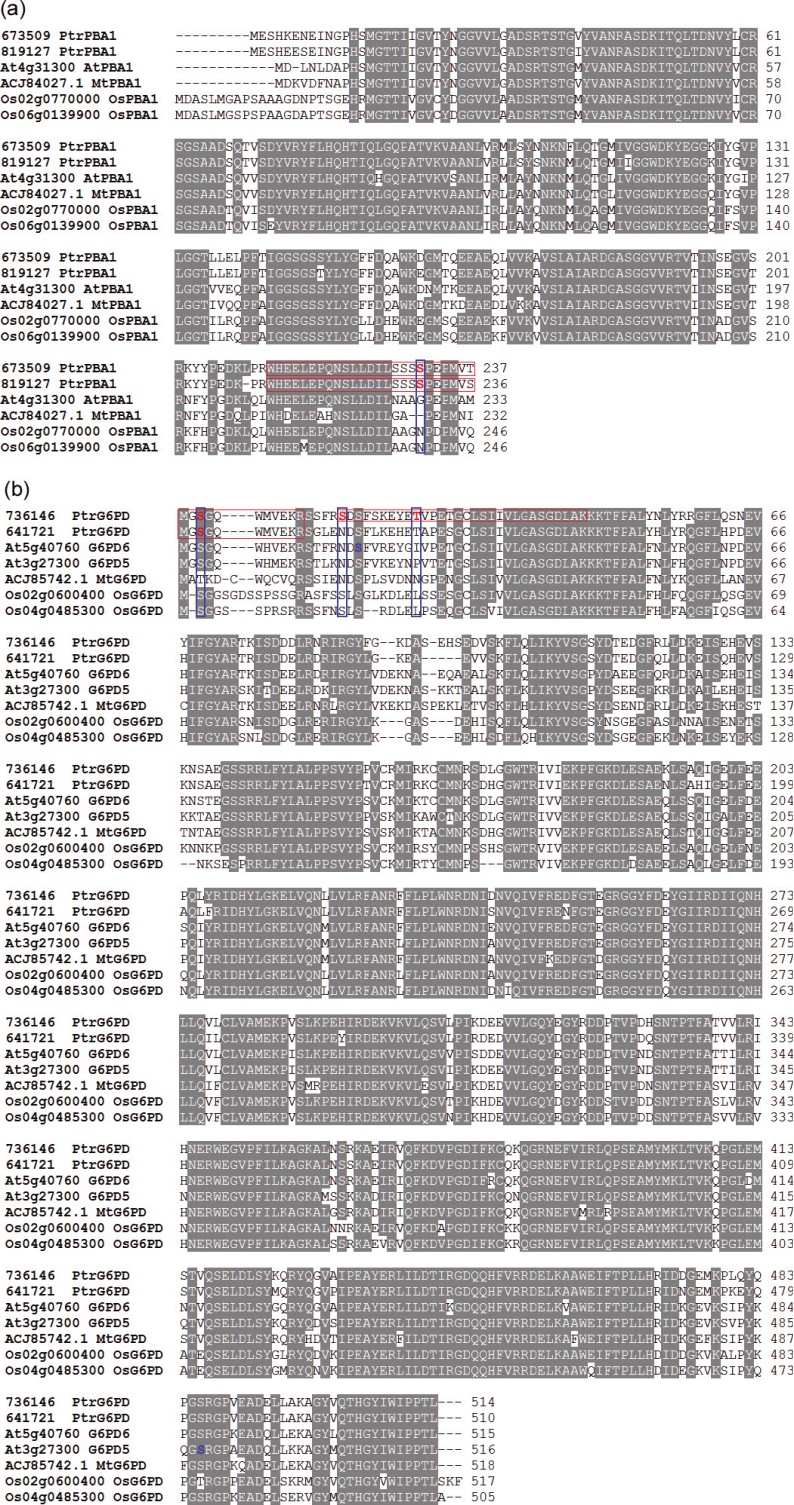
**Sequence alignment of poplar phosphoproteins and their closest *Arabidopsis *homologs to identify unique phosphosites in poplar**. Asterisk indicates phosphorylation site. Fine red boxes show phosphopeptides identified in this study. Phosphorylation sites identified from poplar in our study are shown in red bold font. Blue bold boxes show non-conserved phosphorylation sites. (a) Sequence alignment with all PBA1 orthologs. (b) Sequence alignment with all G6PD orthologs.

Many physiological features of woody plants are not reflected in herbaceous models, e.g., *Arabidopsis *or rice. In our study, several poplar phosphoproteins were highly conserved with their *Arabidopsis *ortholog(s), but their corresponding phosphorylation sites were not conserved (Additional file 9). For example, the poplar 20S proteasome subunit protein (PtrPBA1) shared high sequence similarity with its orthologs in *Arabidopsis *(AtPBA1), *Medicago truncatula *(MtPBA1), and rice (OsPBA1). In PtrPBA1 (673509 and 819127), there is a C-terminal motif that includes a pSer residue at position 231. This motif is conserved across two other PtrPBA1 isoforms (Figure 3a), but the equivalent sites are substituted with a non-phosphorylatable residue in the homologs in the other three species (Figure 3a). The poplar glucose-6-phosphate 1-dehydrogenase isoforms (PtrG6PD, 736146 and 641721) are another good example; they share high sequence similarity with their homologs in *Arabidopsis *(AtG6PD), *M. truncatula *(MtG6PD), and rice (OsG6PD). However, PtrG6PD (736146) is phosphorylated at the N-terminus at residue Thr25 (Figure 3b), which is conserved across poplar G6PD isoforms, but the residues at the equivalent position in G6PD isoforms of *Arabidopsis*, *Medicago*, and rice are non-phosphorylatable. Interestingly, pSer16 is conserved across rice G6PD orthologs, but it is substituted with a non-phosphorylatable Asn residue in its *Arabidopsis *and *Medicago *orthologs (Figure 3b). These findings suggest that there are unique mechanisms regulating phosphorylation in poplar.

In summary, identification of new phosphorylation sites can provide significant biological insights about the cellular mechanisms of signaling activation and inhibition. Although many phosphorylation sites have been identified in *Arabidopsis *from the PhosPhAt database [55], we identified 99 novel phosphosites and 41 novel phosphoproteins in poplar in the present study. These novel phosphoproteins and phosphorylation sites could provide useful data to identify components of phosphorylation-dependent signal cascades, and to determine the function of phosphorylation events in responses to specific environment signals.

### Classification of the DTB phosphoproteome

**Figure 4:**
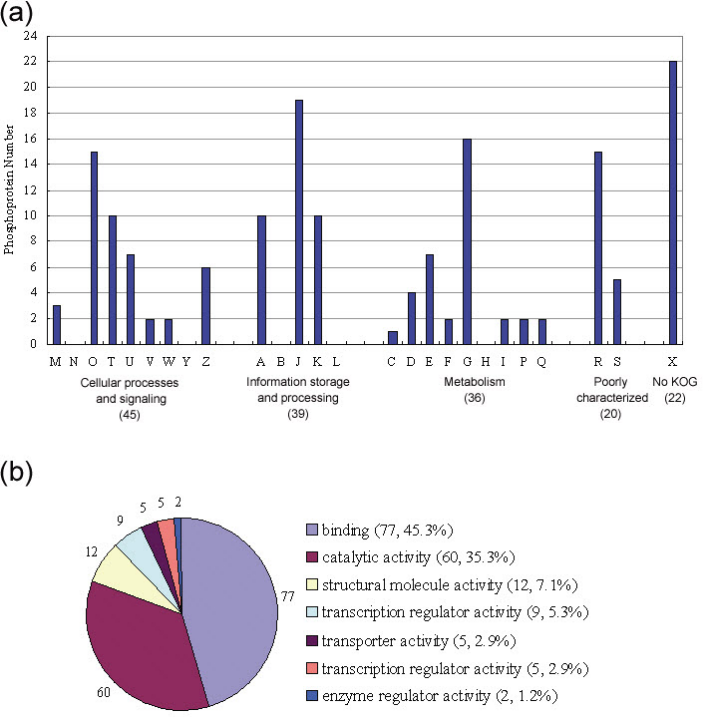
**KOG and molecular functional classification of phosphoproteins identified from poplar DTBs with verified phosphopeptides (*n *= 151)**. (a) KOG classification of phosphoproteins identified from poplar DTBs; X represents phosphoproteins without KOG classification; (b) Molecular functional classification of identified phosphoproteins.

Figure 4a shows the results of a euKaryotic Orthologous Groups (KOG) classification analysis [56] of the 151 phosphoproteins. The KOG classification of the identified phosphoproteins and all proteins encoded in the *P. trichocarpa *genome are shown in Additional files 10 and 11, respectively. Of the 151 phosphoproteins, 129 were assigned a KOG ID according to the KOG classification. The remaining phosphoproteins were poorly annotated and could not be assigned to any KOG group. The classified proteins were further divided into various subgroups: the largest functional subgroup consisted of 19 phosphoproteins, which were assigned to the J subgroup (translation, ribosomal structure, and biogenesis), 16 phosphoproteins were assigned to the G subgroup (carbohydrate transport and metabolism), and 15 phosphoproteins were assigned to the O subgroup (post-translational modification, protein turnover, chaperones) (Figure 4a and Additional file 11).

Functional annotation of phosphoproteins was also conducted using the Blast2Go program [57]. Sequences were searched against the non-redundant (NR) protein database at NCBI. These identified phosphoproteins were categorized into seven major classes with diverse functions (Figure 4b): 80.6% were related to binding affinity (45.3% to binding affinity associated with regulation of gene expression and catalytic activity, and 35.3% to binding affinity related to carbohydrate transport, biosynthesis, and metabolism). The rest were categorized as having structural molecule activity (7.1%), translation (5.3%) or transcription regulator activity (2.9%), membrane proteins with transporter activity (2.9%), and enzyme regulator activity (1.2%) (Figure 4b). In this study, most of the identified phosphoproteins were involved in binding and catalytic activity, consistent with previous studies [22,32,33].

### Potential protein kinases involved in signal transduction during dormancy in poplar

**Figure 5:**
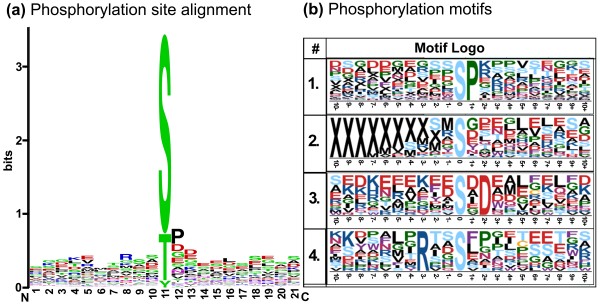
**Sequence alignment of phosphorylation sites and extraction of significantly enriched phosphorylation motifs**. (a) Amino acid sequence around the phosphorylated amino acid based on alignment of all phosphorylation sites from the identified DTBs phosphopeptide dataset using Weblogo. (b) Motif-X-extracted motifs from entire phosphopeptide dataset. JGI *Populus trichocarpa *v1.1 protein database was used as the background database to normalize the score against a random distribution of amino acids. Note that only those phosphorylated amino acids that were confidently identified as the exact site of phosphorylation were used for the analysis (see "Materials and Methods" for detailed description). Motif 1, Pro-directed kinase motif (*n *= 40); Motif 2, Unknown phosphorylation motifs (*n *= 20); Motif 3, CKII motif (*n *= 17); Motif 4, 14-3-3 binding motif (*n *= 13).

Confirmed phosphorylation sites are footprints of kinase activities. To date, several kinases have been documented in *Arabidopsis*, and their substrate spectra and functional interactions have mainly been deciphered by large-scale investigations of phosphoproteins [22,33]. However, little is known about the kinases involved in regulating dormancy in plants. To identify the protein kinases responsible for phosphorylation of the phosphosites identified in this study, we obtained putative phosphorylation motifs from the phosphopeptide dataset using the Motif-X software tool (Figure 5). This tool extracts overrepresented patterns from any sequence dataset by comparing it to a dynamic statistical background [58]. Four significantly enriched phosphorylation motifs were extracted from the identified DTB phosphopeptides dataset (Figure 5b). One of the enriched phosphorylation site motifs resembled a known motif in proline-directed kinases (pS/pTP). This was also supported by the alignment of all the identified DTB phosphorylation sites (Figure 5a). The identity of the second enriched motif was unknown, and had no counterparts in any known kinases. The third enriched phosphorylation motif showed high similarity to a motif found in members of the casein kinase II subfamily (pS/pTXXE/D). Members of this family can phosphorylate a wide variety of plant proteins *in vitro*. The fourth enriched motif was similar to the 14-3-3 binding motif (RXXpS/pT). Kinases with this motif regulate the activities of the vacuolar potassium channel KCO1 and the vacuolar ATPase [59] (Figure 5b). These results suggest that proline-directed kinases could be the major kinase group involved phosphorylation of these identified proteins during dormancy in poplar (Figure 5).

## Discussion

A series of differential expression profiling analyses of the induction, maintenance, and release of bud dormancy made it possible to identify a large set of dormancy-related candidate genes [1,9-12,60-66]. These genes were mainly involved in ABA signaling pathways, cold and oxidative responses, flavonoid biosynthesis, flowering time, and circadian regulation [66,67]. Although there is increasing information available about the roles of genes and their products in dormancy, very little is known about the relevance of protein phosphorylation in dormancy. To address this, in this work, we identified the phosphorylation status of proteins in dormant terminal buds of poplar using mass spectrometry combined with TiO_2 _phosphopeptide-enrichment strategies. However, it remains unknown whether these phosphoproteins identified in dormant buds in this study actually participate in dormancy-related processes. To interpret the significance of the presence of these phosphoproteins in dormant buds, we compared the identified phosphoproteins with previously reported dormancy-related genes and their products. Notably, some of these phosphoproteins were well matched to homologs of known dormancy-related candidate gene-products identified in previous studies of various species. Some of these common proteins of interest are briefly discussed in the context of dormancy.

### Phosphoproteins involved in dormancy-related signal transduction

Abscisic acid (ABA) is the major plant hormone involved in growth, dormancy, and cold acclimation [68]. The ABA signaling pathway is regulated by reversible protein phosphorylation mediated by protein kinases and phosphatases [68]. Genetic evidence demonstrated that sucrose non-fermenting (SNF)-like protein kinase, receptor-like protein kinase (LRK), and protein phosphatases 2C (PP2Cs) encoded by *ABI1 *and *ABI2 *are important regulators of the ABA signaling pathway, which plays an important role in the induction or release of bud dormancy [5,6,10,63,68-72]. In this work, three SNF1-type kinases in poplar (299214, 818055, and 828986) containing the phosphopeptide "DGHFLKTSCGpSPNYAAPEVISGK", and one leucine-rich repeat receptor-like protein kinase (LRK, 422370) were phosphorylated (Additional files 12 and 13). These phosphorylation sites were all well conserved, and corresponding phosphosites were identified in *Arabidopsis *(Additional file 12). In the case of PP2C, the Ser131 in the phosphopeptide "VSGMIEGLIWpSPR" from PP2C (554898, 587195) was identified as a novel phosphorylation site (Additional file 14). Calmodulin (CaM) and the CaM-binding protein play an important role in Ca^2+ ^signaling, which is related to bud dormancy [61,64,70,73,74]. In this study, two CaM family proteins (729432 and 823453) were phosphorylated (Additional file 3 and Additional file 13); however, the corresponding site has not been identified as a phosphorylation site in their respective *Arabidopsis *counterparts, AT1G56340.1 and AT5G61790.1.

### Phosphoproteins involved in auxin responses and growth development related to dormancy

The auxin-sensitive Dormancy-associated/auxin-repressed (DAAR) gene is associated with bud dormancy [66,75,76]. In this study, one DAAR protein (647948) showed three isoforms with respect to phosphorylation status, the three forms respectively phosphorylated at Thr61, Thr63, and Thr70 (Additional file 3 and Additional file 13). These corresponding sites have not been identified as phosphorylation sites in its homolog in *Arabidopsis*, the DAAR protein (AT1G28330.1). Interestingly, the *Arabidopsis *DAAR protein is phosphorylated at its conserved Thr28 and Thr29 residues [33].

Vernalization independence 4 (VIP4) interacts with the FLOWERING LOCUS C-LIKE MADS-BOX PROTEIN (FLC) to activate FLC, leading to inhibition of flower development [77-79]. They are key components in the regulatory pathway of cold-mediated bud dormancy induction and release [4,77]. In our study, we observed that poplar VIP4 (569930) was phosphorylated at Ser225 (Additional file 3 and Additional file 13); the corresponding site in its *Arabidopsis *homolog (AT5G61150.2) is also known to be phosphorylated [50]. The mei2-Like (ML) genes, which play roles in plant meiosis and development [80], were preferentially expressed in dormant buds of leafy spurge [66]. In this study, two phosphorylation sites were respectively identified on the N- and C- terminus of two isoforms of poplar mei2-like proteins (714870 and 410877), which are homologous to *Arabidopsis *ML (AT1G29400.2) (Additional file 3 and Additional file 13). The corresponding site at the N-terminus in *Arabidopsis *ML is known to be phosphorylated [50], while the C-terminal phosphorylation site was novel.

### Phosphoproteins involved in dormancy-related cold stress response

Dehydrins (DHNs) are Group II (D-11 family), late embryogenesis abundant (LEA) proteins that accumulate in response to water deficit induced by drought, low temperature, or salinity [81-84]. Certain DHNs play a vital role in bud dormancy and cold acclimation of trees [1,12,66,85-88]. Phosphorylation of their S-segment is required for targeting to the nucleus [89-91]. In this study, three DHN proteins were phosphorylated in regions outside of the S-segment, one (663123) belongs to the K_n _type of DHNs, one (571250) belongs to the K_n_S type of DHNs, and the other (818850) belongs to the SK_n _type of DHNs (Additional file 3 and Additional file 13). Heat shock proteins (HSP) function as molecular chaperones, and are induced by various environmental stress, such as cold, salinity, and oxidative stress [92]. Recent data suggested that they are also involved in the process of bud dormancy [12,93,94]. A phosphorylation event on an HSP was identified in *Arabidopsis *[22,40]. Here, two HSP70s (657150 and 769322), one HSP90 (652330), and one HSP26 (832078) were phosphorylated in poplar (Additional file 3 and Additional file 13).

### Phosphoprotein associated with dormancy-related flavonoid biosynthesis

Many genes related to flavonoid biosynthesis are significantly regulated during the release of dormancy, such as acetyl-CoA carboxylase (ACCase), chalcone synthase, chalcone isomerase, and flavonol synthase [12,65-67]. Acetyl-CoA carboxylase (ACCase) catalyzes the formation of malonyl-CoA, which is the substrate for biosynthesis of fatty acids and secondary metabolites, such as flavonoids and anthocyanins [67]. In this work, one putative ACCase (736443) was phosphorylated at Ser94 and Ser95 (Additional file 3 and Additional file 13). There have been no reports of phosphorylation of its homolog in *Arabidopsis *(AT5G16390.1). Interestingly, we also found another phosphorylation event related to flavonoid biosynthesis; polyphenol oxidase (PPO) (275859) was phosphorylated at Ser452 (Additional file 3 and Additional file 13). The poplar PPO has no counterparts in *Arabidopsis*, but it shows homology to aureusidin synthase (AS) in *Antirrhinum majus*, a flavonoid synthase enzyme that catalyzes the formation of aurones from chalcones [95]. To our knowledge, this is the first report of a specific phosphorylation site in a plant flavonoid synthase. The existence of this site suggests that phosphorylation may regulate its functions.

### Phosphoproteins involved in transport related to dormancy

The plasma membrane H+-ATPase (AHA) is responsible for the transport of protons out of the cell through the membrane [96]. The AHA gene is strongly expressed during dormancy transition, and contributes to changes in the plasma membrane [12]. The regulation of AHA is controlled by phosphorylation of one Thr residue in the well-conserved C-terminal domain [97,98]. In the AHA family in *Arabidopsis*, the well-conserved Thr residue is phosphorylated in response to stress [37,42,97]. Here, the exact Thr site (Thr949) in the C-terminus of poplar AHA10 (826518), and its corresponding site in AHA11 of poplar (422528) were both phosphorylated (Figure 2a). Another example of a transport protein is ATP-binding cassette (ABC) transporters, which are integral membrane proteins that transport a wide variety of substrates, such as ABA, auxin, and some plant secondary metabolites across cellular membranes [99,100]. Genes encoding ABC transporters are regulated during dormancy transition [11,12,66], suggesting that they are linked with dormancy. Here, two ABC transporter family proteins (554850 and 800153) were phosphorylated at Thr55 (Additional file 3 and Additional file 13). The corresponding site is phosphorylated in its homologs in rice, *Arabidopsis*, and *Medicago *[42,49,50].

### Phosphoproteins involved in protein synthesis related to dormancy

Some genes and proteins involved in protein biosynthesis play a role in the mechanism of bud dormancy release [12,60,101]. Phosphorylation of ribosomal proteins can affect protein synthesis by altering ribosome structure [45]. In the present work, six 60S acidic ribosomal proteins including P0-, P1-, P2-, and P3-types were phosphorylated close to their conserved C terminus, consistent with results reported elsewhere [45]. However, the pSer at position 2 on the 40S ribosomal protein S12 of poplar (RPS12, 714910) was novel (Additional file 15). Recent evidence suggests that phosphorylation of Ser2 plays an important role in regulating nucleocytoplasmic shuttling of eukaryotic translation initiation factor 5A (eIF5A) in plant cells [102-104]. Here, four poplar eIF5A proteins (717121, 832646, 835953, and 724093) were phosphorylated at their well-conserved serine residue and acetylated at their N-terminus (Additional file 16). Phosphorylation regulates the function and/or location of translation elongation factor 1A (eEF1A), which is involved in protein biosynthesis and signal transduction [105-107]. Here, five eEF1A isoforms (256777, 655943, 675976, 655949, and 720367) from poplar, all containing the phosphopeptide pSVEMHHEALQEALPGDNVGFNVK (Ser279) were novel phosphoproteins (Additional file 17).

### Phosphoproteins involved in electron transport or energy pathways

There are increases in expressions of some genes involved in energy pathways during bud release, including glyceraldehyde-3-phosphate dehydrogenase (GAPC) and phosphoenolpyruvate carboxylase (PEPC) [11,12,60,93]. Here, three GAPC isoforms (821843, 575307 and 728998) and three PEPC isoforms (552645, 745223, and 728315) were phosphorylated (Additional file 13 and Additional file 3). The light harvesting complex protein Lhcb1, which is essential for light electron transport, is significantly regulated during bud release [11,63,66]. Reversible phosphorylation of Lhcb1 is important for distributing absorbed light energy between the two photosystems [108,109]. As reported in other experiments on *Arabidopsis *[33,110] and spinach, Lhcb1 proteins are phosphorylated at several Thr and Ser residues in their amino terminus [108]. Here, we identified two previously unknown phosphorylation sites on the poplar Lhcb1 protein; the conserved Thr38 phosphosite and the unconserved Thr39 phosphosite (Figure 2b).

In summary, this information on phosphoproteins in dormant poplar provides a useful dataset, and provides new insights for exploring the relevance of phosphorylation for dormancy. However, further research, e.g., comparing proteomes between dormant/non-dormant tissues, is required to clarify the roles of phosphorylation in the dormancy process.

## Conclusions

Many physiological features of woody plants are not reflected in the herbaceous model *Arabidopsis *or in rice. Therefore, it is important to determine phosphorylation sites in poplar proteins, and to determine the roles of these phosphorylations in modifying protein function during growth and development. To date, there have been no extensive studies on the poplar phosphoproteome. In this work, we conducted a detailed analysis of the phosphoproteome of dormant poplar buds using an MS method and TiO_2 _phosphopeptide-enrichment strategies. We found 161 unique phosphorylated sites in 161 phosphopeptides from 151 proteins, most of which are associated with binding and catalytic activity. Most of the poplar phosphoproteins have orthologs in *Arabidopsis*, suggesting that there are similar signaling pathways mediated by phosphorylation in poplar and *Arabidopsis*. However, some phosphoproteins and phosphorylated sites were unique to poplar, thus confirming the need to obtain phosphoproteome data from poplar. Several phosphorylation motifs were extracted from the dataset by Motif-X. This could provide evidence for the involvement of kinases in phosphorylation of these identified proteins during dormancy in poplar. Further experiments are now required to confirm that these specific kinases interact with the identified phosphoproteins *in vivo*. A promising way forward is to comprehensively characterize and analyze the dynamics of phosphorylation of poplar proteins in response to environmental changes, using specialized targeted quantitative proteomics tools.

## Methods

### Plant materials and chemicals

Dormant terminal buds were collected from hybrid poplar (*Populus simonii × P. nigra*) in Harbin, China, (E126°37', N45°42') at the end of December, 2009. Samples were frozen in liquid nitrogen and stored at -80°C until use.

Iodoacetamide (IAA) and dithiothreitol (DTT) were purchased from Acros Organics (Morris Plains, NJ, USA). HPLC-grade acetonitrile (ACN) was obtained from JT Baker (Thomas Scientific, Swedesboro, NJ, USA). HPLC-grade water was prepared using a Milli-Q A10 system from Millipore (Billerica, MA, USA). ModiWed sequencing-grade trypsin was supplied by Promega (Madison, WI, USA). Protease-inhibitor cocktail and the 2-D Quant kit were obtained from Amersham Pharmacia Biotech (Uppsala, Sweden). All other reagents were purchased from Sigma (St Louis, MO, USA).

### Preparation of total proteins

The dormant terminal buds were crushed into a fine powder in liquid nitrogen and resuspended at -20°C in 10% (w/v) trichloroacetic acid (TCA) in cold acetone containing 0.07% (v/v) 2-mercaptoethanol for at least 2 h. The mixture was centrifuged at 10000 *g *at 4°C for 1 h, and the precipitates were washed with cold acetone containing 0.07% (v/v) 2-mercaptoethanol. The pellets were dried by vacuum centrifugation and dissolved in 7 M urea, 2 M thiourea, 20 mM dithiothreitol, 1% (v/v) protease-inhibitor cocktail, 0.2 mM Na_2_VO_3_, and 1 mM NaF at room temperature for 2 h, before centrifugation at 40000 *g *at 10°C for 1 h. The resulting supernatant was collected and kept at -80°C until further use. The total protein content of the samples was quantified using a 2-D Quant kit.

### In-solution protein digestion

Total proteins were digested as described elsewhere [111,112]. Briefly, the total protein solution was adjusted to pH 8.5 with 1 M ammonium bicarbonate. Then, the sample was reduced for 45 min at 55°C by adding DTT to a final concentration of 10 mM, and then carboxyamidomethylated by incubation with 55 mM IAA for 30 min in the dark at room temperature. After this step, CaCl_2 _was added to a final concentration of 20 mM. Then, endoprotease Lys-C was added to a final substrate-to-enzyme ratio of 100:1, and this reaction was incubated for 12 h at 37°C. The Lys-C digest was added to 1 M urea containing 100 mM ammonium bicarbonate, and modified trypsin was added to a final substrate-to-enzyme ratio of 50:1. The trypsin digest was also incubated at 37°C for 12 h. After digestion, the peptide mixture was enriched using TiO_2 _microcolumns for further MS analysis.

### Enrichment of phosphorylated peptides using TiO_2 _microcolumns

The TiO_2 _microcolumns were packed as described elsewhere [25]. A small plug of C8 material was stamped out of a 3M Empore C8 extraction disk with a HPLC syringe needle and placed to form a frit at the small end of the GELoader tip. The TiO_2 _beads were suspended in 100% ACN, and an appropriate volume of this suspension (depending on the size of the column) was loaded into the GELoader tip. Gentle air pressure produced by a plastic syringe was applied to pack the column. The TiO_2 _microcolumn was equilibrated with loading buffer (40 μl; 80% ACN/5% TFA/saturated phthalic acid solution). Immediately, the trypsin-digested peptide mixture diluted in loading buffer was added to the TiO_2 _microcolumn. Then, the column was washed once with loading buffer (40 μl) and three times with washing buffer (40 μl; 80% ACN/2% TFA). The washing and loading buffer contained 80% ACN organic solvent in order to abrogate the adsorption of peptides to the C8 material [28]. The bound peptides were eluted twice with 40 μl ammonium bicarbonate (pH > 10.5), and then with 10 μl 30% ACN. The eluted phosphopeptides were lyophilized and then dissolved in 1% formic acid before MS analysis.

### NanoUPLC-ESI-MS/MS

NanoUPLC-ESI-MS/MS was performed with a splitless nanoUPLC (10 kpsi nanoAcquity; Waters) in combination with a Synapt high-definition mass spectrometer with a nanospray ion source (Waters). A symmetric C_18 _5-μm, 180-μm × 20-mm pre-column and a BEH C_18 _1.7-μm, 75-μm × 250-mm analytical reversed-phase column (Waters) were used. The MassLynx (version 4.1; Waters) program was used for instrument control and data acquisition. The mobile phases were (A) 100% H_2_O/0.1% formic acid and (B) 100% ACN/0.1% formic acid. The samples were dissolved in aqueous 0.1% formic acid solution and loaded onto the pre-column at a flow rate of 5 μl/min for 3 min. The phosphopeptides were separated by a gradient of 5-40% mobile phase B for 90 min at a flow rate of 200 nl/min, followed by a 10-min rinse with 90% mobile phase B. The column was re-equilibrated with the initial conditions for 20 min. The lock mass was delivered from the auxiliary pump of the NanoAcquity pump at a constant flow rate of 400 nl/min at a concentration of 100 fmol/μl of (Glu1) fibrinopeptide B to the reference sprayer of the NanoLockSpray source from the mass spectrometer. In this study, every sample was analyzed in triplicate. Data-dependent acquisition was carried out in positive ion mode. MS spectra were acquired for 1 s from mass-to-charge ratios of (*m/z*) 350 to 1990. Two of the most intense precursor ions that were doubly or triply charged were selected from *m/z *350 to 1990. MS/MS spectra produced by collision-induced dissociation (CID) were acquired for 2 s from *m/z *50 to 1990. The collision energy was automatically calculated according to peptide charge and *m/z*; a dynamic exclusion window was applied to prevent the same *m/z *from being selected for 2 min after its acquisition. The candidate phosphopeptides were initially assigned by ESI-MS/MS using 79.96-Da mass increments per phosphate moiety relative to the unmodified peptides. To detect the phosphopeptides, we utilized the preferred loss of the phosphate group upon collision-induced dissociation. In positive ion tandem MS, an intense neutral loss of 98 Da, corresponding to H_3_PO_4_, was observed for peptides containing phosphorylated Ser, Thr, and Tyr residues.

### Data analysis and Mascot database search

The MS/MS data were processed and converted to a pkl file format with ProteinLynx software (Waters), and the resulting pkl file was used to search against the JGI *Populus trichocarpa *v1.1 (http://genome.jgi-psf.org/Poptr1_1/Poptr1_1.home.html) protein sequence database using an in-house Mascot server (version 1.8) with acetylation in the N-terminus of the protein, carbamidomethylation, methionine oxidation, and phosphorylation of serine/threonine/tyrosine residues as variable modifications. Two missed cleavage sites were allowed. The search was performed with a peptide mass tolerance of 15 ppm in the MS and 50 ppm in the MS/MS modes. The false discovery rate (FDR) was 0.00% for peptide matches above the identity threshold and 0.36-0.85% for peptide matches above the homology or identity threshold.

### Bioinformatics

Using a custom Perl program, all the phosphoprotein sequences were extracted from protein databases (http://genome.jgi-psf.org/Poptr1_1/Poptr1_1.home.html) by their protein ID. The Blast2Go program [57] was used to obtain descriptions of protein sequences by a BlastP search against a non-redundant protein database (http://blast.ncbi.nlm.nih.gov/Blast.cgi) with default parameter settings. Protein functions, annotations, and classifications were also examined using gene ontology (GO), GO-EnzymeCode, and InterPro databases and search tools.

The Batch sequence search tool (http://pfam.sanger.ac.uk/search) was applied to obtain Pfam information for identified phosphoproteins. The significantly enriched phosphorylation motifs set was extracted from our phosphopeptide data using the Motif-X algorithm [58]. All phosphorylated peptides with confidently identified phosphorylation sites were used as the data set to extract significantly enriched phosphorylation motifs. The phosphopeptides were centered at the phosphorylated amino acid residues and aligned, and ten positions upstream and downstream of the phosphorylation site were included. In the case of C- and N-terminal peptides, the sequence was completed to 21 amino acids with the required number of "X"s, where X represents any amino acid. As the background data set, protein sequences of the entire genome poplar database *Populus trichocarpa *v1.1 in Fasta format (in a shortened version due to upload restrictions of 10 MB) were used. The occurrence threshold was set to 5% of the input data set at a minimum of three peptides, and the probability threshold was set to P <10^-5^. Amino acid sequences around the phosphorylated amino acid based on the alignment of all the phosphorylation sites were completed by the Weblogo program [113] in the entire identified DTBs data set.

## List of abbreviations

**DTB**: Dormant terminal buds; **NanoUPLC**: Nano ultra-performance liquid chromatography; **Ser**: Serine; **Thr**: Threonine; **Tyr**: Tyrosine; **PTM**: Post-translational modification.

## Competing interests

The authors declare that they have no competing interests.

## Authors' contributions

The study was conceived by CPY and ZGW. CCL and HXW carried out experimental work, participated in data analyses, and drafted the manuscript. CFL and ZYS participated in the design of the study and performed *in silico *analyses. All authors read and approved the final manuscript.

## Supplementary Material

Additional file 1 **Nine sheets as follows**: Sheet 1: Contents. Sheet 2: Phosphopeptide identification list. Sheet 3: Phosphorylation site list. Sheet 4: Blast results. Sheet 5:Annotation_of_phosphoproteins. Sheet 6: KOG classifications. Sheet 7: Pfam_domain_information. Sheet 8: Source_for_motif_analysis. Sheet 9: pS_motifs. Click here for file

Additional file 2 **Phosphopeptides and phosphorylation sites identified in dormant terminal buds of poplar**. Click here for file

Additional file 3 **Detailed information for phosphopeptides and phosphoproteins identified in dormant terminal buds of poplar**. Click here for file

Additional file 4 **MS/MS spectra (in a separate file). File contains all the original MS/MS spectra of 161 phosphopeptides identified in this study**. Click here for file

Additional file 5 **Comparison of singly and doubly phosphorylated peptides**. Click here for file

Additional file 6 **Location of phosphorylation sites in characterized conserved domains**. Click here for file

Additional file 7 **Flowchart for analyzing the conservation of phosphoproteins and phosphosites between poplar and *Arabidopsis***. Click here for file

Additional file 8 **Conserved phosphorylation sites within orthologous proteins**. (a) Phosphosites conserved in orthologous proteins. (b) Phosphosites that were not conserved in orthologous proteins. Click here for file

Additional file 9 **Unconserved phosphorylation sites within orthologous proteins**. Click here for file

Additional file 10 **KOG analysis of identified phosphoproteins and all proteins encoded in *Populus trichocarpa *genome**. (a) Percentage of KOG functional group categories from the identified phosphoproteins and all proteins encoded in *Populus trichocarpa *genome. (b) Percentage of KOG functional subgroup categories from the identified phosphoproteins and all proteins encoded in *Populus trichocarpa *genome. Click here for file

Additional file 11 **Complete list of KOG analysis of phosphoproteins and all proteins encoded in *Populus trichocarpa *genome**. Click here for file

Additional file 12 **Sequence alignment of phosphorylated sites in protein kinases between poplar and *Arabidopsis***. Click here for file

Additional file 13 **Detailed information for identified phosphoproteins referred to in discussion section**. Click here for file

Additional file 14 **Sequence alignment of phosphorylated sites in protein phosphatases between poplar and *Arabidopsis***. Click here for file

Additional file 15 **Sequence alignment of RPS12 between poplar and *Arabidopsis***. Click here for file

Additional file 16 **Sequence alignment of conserved N-terminus of eIF5A between poplar and *Arabidopsis***. Click here for file

Additional file 17 **Sequence alignment of conserved C-terminus of EF-1-alpha between poplar and *Arabidopsis***. Click here for file
