# Supplementary material for: Identification and analysis of phosphorylation status of proteins in dormant terminal buds of poplar
Source: BMC Plant Biol. 2011 Nov 11;11:158. doi: 10.1186/1471-2229-11-158 (PMC3234192; doi:10.1186/1471-2229-11-158)
Supplement: Additional file 2 — Phosphopeptides and phosphorylation sites identified in dormant terminal buds of poplar. [file 1471-2229-11-158-S2.DOC]

| **Additional file 2** Phosphopeptides and phosphorylation sites identified in phosphoproteome of dormant terminal buds of poplar | | | | | | | | | | |
| --- | --- | --- | --- | --- | --- | --- | --- | --- | --- | --- |
| Protein ID | | Annotation | Identified Phosphopeptides^(c)^ | | Res. Pos. | AGI code | Simi. (%) | | Identified phosphopeptides from PhosphAT Database | Ref. |
| **1. Cellular Processes and Singnaling** | | | | | | | | | | |
| *Cell wall/membrane/envelope biogenesis (M)* | | | | | | | | | | |
| 835143 | GDP-mannose pyrophosphorylase | | RISS*FEALQSATR | | S217 | At1g74910 | 95.2 | RVSS*FEALQPATR | | (Sugiyama et al., 2008) |
| 828302 | glycosyl transferase family protein^(b)^ | | TQ**T***AGNLGESMLDSEVVPSSLVEIAPILR | | T21 | At1g05570 | 87.0 | UI | |  |
| 578888 | Glycosyltransferase | | INS*VDAMEAWVNQQK | | S56 | At5g20280 | 85.1 | INS*AESMELWASQQK | | (Reiland et al., 2009) |
|  |  |  | EAVADMS*EDLSEGEKGDTVGDLSAHGDSVR | | S133 |  |  | EATADMS*EEFSEGEK | | (Sugiyama et al., 2008) |
|  |  |  | EAVADMS*EDLS*EGEKGDTVGDLSAHGDSVR | | S137 |  |  | EATADMSEEFS*EGEK | | (Sugiyama et al., 2008) |
| *Cytoskeleton (Z)* | | | | | | | | | | |
| 263289 | Alpha tubulin | | TVQFVDWCPT*GFK | | T174 | At5g19770 | 97.8 | TVQFVDWCPT*GFK | | (Sugiyama et al., 2008) |
| 571195 | Tubulin alpha-6 chain | |  |  | T349 | At5g19780 | 98.5 |  |  |  |
| 814768 | Tubulin alpha-4 chain | |  |  | T349 | At5g19780 | 98.0 |  |  |  |
| 826146 | Tubulin alpha-8 chain | |  |  | T349 | At5g19770 | 98.4 |  |  |  |
| 831020 | Tubulin alpha-2 chain | |  |  | T349 | At5g19780 | 98.4 |  |  |  |
| 550223 | GAS (Growth-arrest-specific protein), and related proteins | | ILAETQMASES**S***PHHYR | | S589 | At5g55860 | 76.2 | UI | |  |
| 552310 | von Willebrand factor and related coagulation proteins | | AVTTNATRVP**S***PGPSLR | | S350 | At3g19430 | 41.7 | UI | |  |
|  |  |  | GAFMEVIR**S***PK | | S215 |  |  | UI | |  |
| 572436 | von Willebrand factor and related coagulation proteins | |  |  | S340 | At3g22120 | 39.8 | UI | |  |
| *Defense mechanisms (V)* | | | | | | | | | | |
| 552310^(a)^ |  | |  | |  |  |  |  | |  |
| 572436^(a)^ |  | |  | |  |  |  |  | |  |
| *Intracellular trafficking, secretion, and vesicular transport (U)* | | | | | | | | | | |
| 573860 | Cytosolic sorting protein GGA2/TOM1 | | MSAGMSSMS*FK | | S35 | At5g16880 | 84.8 | ISAGVSSMS*FK | | (Reiland et al., 2009) |
| 827509 | Cytosolic sorting protein GGA2/TOM1 | |  |  | S35 | At5g16880 | 82.3 |  |  |  |
| 743875 | Vacuolar sorting protein VPS1, dynamin, and related proteins | | HSLS*DGSLDTMAR | | S714 | At1g59610 | 87.2 | QSLS*EGSLDK | | (Sugiyama et al., 2008) |
| 821436 | Vesicle coat complex COPII, subunit SEC31 | | LDDTPADEMGYENNQEATIFSADDGEDFFNNLP**S***PK | | S523 | At3g63460 | 78.9 | UI | |  |
| 798347 | Synaptic vesicle protein EHS-1 and related EH domain proteins | | FDSFSMNEGGF**S***PR | | S876 | At1g20760 | 63.2 | UI | |  |
| 797875 | ER-Golgi vesicle-tethering protein p115 | | LMELGEDVDKLLEGVGDDMGLPEDS*EKEED | | S910 | At3g27530 | 86.0 | LLEDIGDESEAQAES*EED | | (Reiland et al., 2009) |
| 774347 | Rab6 GTPase-interacting protein involved in endosome-to-TGN transport | | GYV**S***GDEAVIEEQK | | S383 | At4g00640 | 54.2 | UI | |  |
| *Posttranslational modification, protein turnover, chaperones (O)* | | | | | | | | | | |
| 835143^(a)^ | GDP-mannose pyrophosphorylase | |  | |  |  |  |  | |  |
| 657150 | Molecular chaperones HSP70/HSC70, HSP70 superfamily | | LMPEPTAVALLYAQQQQQTVHENMGSG**S***EK;  LMPEPTAVALLYAQQQQQTVHENMG**S***GSEK | | S224 | At2g32120 | 88.2 | UI | |  |
|  |  |  |  |  | S222 |  |  | UI | |  |
| 769322 | Molecular chaperones HSP70/HSC70, HSP70 superfamily | |  |  | S224 |  | 87.5 |  | |  |
|  |  |  |  |  | S222 |  |  |  | |  |
| 652330 | Molecular chaperone (HSP90 family) | | EIS*DDEDEDVEDKKDEEGNVEDVDDEK | | S219 | At5g56030 | 96.3 | EIS*DDEEEEEK | | (Li et al., 2009) |
|  |  |  | **T***TEKEISDDEDEDVEDKKDEEGNVEDVDDEK | | T213 |  |  | UI | |  |
| 832078 | Molecular chaperone (small heat shock protein Hsp26/Hsp42) | | IMGLDAPLFNTLQHMMDA**S***DHEADKSFNAPTR | | S23 | At5g12020 | 84.7 | UI | |  |
| 830227 | Multifunctional chaperone (14-3-3 family) | | DNLTLWTSDLS*EEGGEHSTAVEPR | | S237 | At2g42590 | 90.0 | DNLTLWTSDI(pS)EEGGDDAHK | | (Reiland et al., 2009) |
| 673509 | 20S proteasome beta subunit A-1 | | WHEELEPQNSLLDILSSS**S***PEPMVT | | S231 | At4g31300 | 94.3 | UI | |  |
| 819127 | 20S proteasome beta subunit A-1 | | WHEELEPQNSLLDILSSS**S***PEPMVS | | S231 |  | 94.7 | UI | |  |
| 823453 | Calnexin | | EG**S***SSGDEKKEETEAENEAAAPAR | | S509 | At5g61790 | 85.9 | UI | |  |
|  |  |  | EGSS***S***GDEKKEETEAENEAAAPAR | | S510 |  |  | UI | |  |
|  |  |  | EGS*SS*GDEKKEETEAENEAAAPAR | | S511 |  |  | S*GDEAEKKEETAAPR | | (Reiland et al., 2009) |
| 729432 | Calreticulin | | KRDEEESKEDPADS*DAEEEDEAGDAEGEDSDAETK | | S379 | At1g56340 | 89.0 | DAPAES*DAEEEAEDDDNEGDDSDNESK | | (Reiland et al., 2009) |
| 834953 | Ubiquitin-like proteins | | **S***GVTGQPQEEDKKPNDQSAHINLK | | S2 | At4g26840 | 94.4 | UI | |  |
| 711526 | Ubiquitin-like proteins | | SGA**T***GQPQEEDKKPNDQSAHINLK | | T5 | At4g26840 | 94.4 | UI | |  |
|  |  |  | **S***GATGQPQEEDKKPNDQSAHINLK | | S2 |  |  | UI | |  |
| 817608 | Ubiquitin-specific protease | | TQ**S***FIPSELSDIFGGQLR | | S104 | At4g30890 | 83.7 | UI | |  |
| 740524 | Anaphase promoting complex, Cdc20, Cdh1, and Ama1 subunits | | #SD**S***ETTPTVSTTAPPSDDHSSAEGIKK | | S4 | At1g47200 | 63.1 | UI | |  |
| 572716 | Heat shock factor binding protein | | EGHDSEDPKQSTADM**T***AFVQHLLQQMQSR | | T17 | At4g15802 | 97.6 | UI | |  |
| *Signal transduction mechanisms (T)* | | | | | | | | | | |
| 798347^(a)^ |  | |  | |  |  |  |  | |  |
| 554898 | Protein phosphatase 2C^(b)^ | | VSGMIEGLIW**S***PR | | S131 | At4g31860 | 84.0 | UI | |  |
| 587195 | Protein phosphatase 2C^(b)^ | |  |  | S131 | At2g25070 | 84.7 |  |  |  |
| 299214 | SNF1 kinase ^(b)^ | | DGHFLKTSCGS*PNYAAPEVISGK | | S179 | At3g29160 | 96.1 | DGHFLKTSCGS*PNYAAPEVISGK | | (Li et al., 2009) |
| 818055 | SNF1 kinase ^(b)^ | |  |  | S179 |  | 87.0 |  |  |  |
| 828986 | SNF1 kinase ^(b)^ | |  |  | S84 |  | 86.7 |  |  |  |
| 422370 | Leucine-rich repeat receptor-like protein kinase (LRK)^(b)^ | | VFDLEDLLRAS*AEVLGK | | S336 | At1g48480 | 79.9 | VFDLEDLLRAS*AEVLGK | | (Nühse et al., 2004) |
| 835719 | UDP-glucose/GDP-mannose dehydrogenase | | KFDWDHPLHLQPKS*PK | | S393 | At3g29360 | 94.4 | FDDHPLHLQPMS*PTTVK | | (Whiteman et al., 2008) |
| 736382 | Ca^2+^-binding protein, EF-Hand protein superfamily | | SQHEKPSYGDD**S***PKRVSHEGGDYERPSYGSR | | S291 | At3g29075 | 44.8 | UI | |  |
| 570193 | Neoplastic transformation suppressor | | LLDTDGESHIDRSDPNYD**S***GEEPYQLVGATISDPIDDYKK | | S110 | At5g63190 | 85.7 | UI | |  |
| **2. Information Storage and Processing** | | | | | | | | | | |
| *RNA processing and modification (A)* | | | | | | | | | | |
| 414256 | Splicing factor 1/branch point binding protein (RRM superfamily) | | | MLQSGLPLDDRPEGAR**S***P**S***PEPIYDNMGIR | S125 | At5g51300 | 79.8 | UI | |  |
|  |  |  |  |  | S127 |  |  | UI | |  |
| 423773 | Splicing factor 1/branch point binding protein (RRM superfamily) | | |  | S133 |  | 80.0 |  | |  |
|  |  |  |  |  | S135 |  |  |  | |  |
| 253138 | Splicing factor RNPS1, SR protein superfamily | | | VEKEMGAVQD**S***PGASATQAEKEEVDSR | S75 | At5g51120 | 81.3 | UI | |  |
| 279975 | Splicing factor RNPS1, SR protein superfamily | | |  | S75 |  | 80.8 |  |  |  |
| 825640 | K-homology type RNA binding proteins | | | RQ**T***GFSSPISDPAPPPSYNSVPPPVDEIQMAK | T37 | At2g25970 | 75.7 | UI | |  |
|  |  |  |  | RQTGFS**S***PISDPAPPPSYNSVPPPVDEIQMAK | S41 |  |  | UI | |  |
|  |  |  |  | RQTGF**S***SPISDPAPPPSYNSVPPPVDEIQMAK | S40 |  |  | UI | |  |
| 653922 | K-homology type RNA binding proteins | | | RQ**T***GFSSPISDPAPPPSYNSVAPPADEIQMAK | T37 | At2g25970 | 73.3 | UI | |  |
|  |  |  |  | QTGFSSPI**S***DPAPPPSYNSVAPPADEIQMAK | S44 |  |  | UI | |  |
|  |  |  |  | RQTGF**S***SPISDPAPPPSYNSVAPPADEIQMAK | S40 |  |  | UI | |  |
|  |  |  |  | RQTGFS**S***PISDPAPPPSYNSVAPPADEIQMAK | S41 |  |  | UI | |  |
| 781843 | K-homology type RNA binding  proteins | | | KLEDLEPETLEEAEPSPADEQEPEEDGKADDVEDGG**S***PDSKR | S57 | At4g10070 | 70.6 | UI | |  |
| 729865 | PolyC-binding proteins alphaCP-1 and related KH domain proteins | | | IIDGLDSDSSN**T***PPTSGAK | T81 | At3g04610 | 81.7 | UI | |  |
| 552542 | ATP-dependent RNA helicase | | | AVDAGMLEYDS*DDNPVVVDKK | S157 | At2g47330 | 82.5 | AVDAGMLDYDS*DDNPIVVDK | | (Jones et al., 2009) |
| 826955 | ATP-dependent RNA helicase | | | EHGRGD**S***PAKSDLDGLTPFEK | S39 | At1g55150 | 90.8 | UI | |  |
| *Transcription (K)* | | | | | | | | | | |
| 572716^(a)^ | Heat shock factor binding protein | | |  |  |  |  |  | |  |
| 826637 | CREB/ATF family transcription factor | | | SKGSLG**S***LNMITGK | S145 | At2g35530 | 76.9 | UI | |  |
| 757220 | CREB/ATF family transcription factor | | |  | S145 |  | 75.9 |  |  |  |
| 663123 | Dehydrin | | | LPGGGGGMTQGG**Y***NQQEHRGGAQGGYNQQEHR | Y115 | AT1G54410 | 57.2 | UI | |  |
| 571250 | Dehydrin | | |  | Y115 | AT1G54410 | 57.2 |  | |  |
| 835251 | Transcriptional coactivator p100 | | | RGLWVHGDIES*DDEDVLPVKK | S969 | At5g07350 | 84.0 | IGIWQYGDIES*DDEDTGPARKPA | | (Reiland et al., 2009) |
| 738421 | CCR4-NOT transcriptional regulation complex, NOT5 subunit | | | SS**S***PSLVDTGLAR | S395 | At5g18230 | 73.4 | UI | |  |
| 717176 | Transcription factor MEIS1 and  related HOX domain proteins | | | NQSGFSFMGSSELDGITQG**S***PKKPR | S351 | At2g35940 | 69.3 | UI | |  |
| 563417 | Transcriptional regulators binding to the GC-rich sequences | | | AAAPDYISLDSGSNHQGGF**S***DEEPEFR | S259 | At5g08550 | 49.2 | UI | |  |
| 649767 | Calcium-responsive transcription coactivator | | | LFLFPVNP**S***PASFGSDGGR | S149 | At5g64430 | 59.5 | UI | |  |
| 247052 | CCAAT-binding factor, subunit A (HAP3) | | | #AD**S***DNES*GGHNAVSELSAKEQDRFLPIANVSR | S4 | At4g14540 | 84.7 | UI | |  |
|  |  |  |  | ADS*DNE**S***GGHNAVSELSAK | S8 |  |  | UI | |  |
| *Translation, ribosomal structure and biogenesis (J)* | | | | | | | | | | |
| 832583 | 60S acidic ribosomal protein P0A^(b)^ | | | KEEPAEES*DDDMGFSLFD | S312 | At3g09200 | 92.0 | VEEKEES*DEEDYGGDFGLFDEE | | (Reiland et al., 2009) |
| 822404 | 60S acidic ribosomal protein P0B^(b)^ | | |  | S310 | At3g09200 | 92.0 |  |  |  |
| 552351 | 60S acidic ribosomal protein P1D^(b)^ | | | KKEEVKEES*EDEDMGFSLFD | S99 | At1g01100 | 76.8 | KKDEPAEES*DGDLGFGLFD | | (Sugiyama et al., 2008) |
| 832971 | 60S acidic ribosomal protein P2B^(b)^ | | | KEEKVEEKEES*DDDMGFSLFD | S103 | At2g27720 | 81.9 | EES*DDDMGFSLFE | | (Carroll et al., 2008) |
| 836661 | 60S acidic ribosomal protein P2C^(b)^ | | |  | S104 | At2g27720 | 79.3 |  |  |  |
| 723244 | 60S acidic ribosomal protein P3A^(b)^ | | | KKEEEPES*DDDMGFSLFD | S106 | At4g25890 | 77.3 | KKEES*EEEEGDFGFDLFG | | (Jones et al., 2009) |
| 714910 | 40S ribosomal protein S12 | | | **S***GEEGAVPQNETPAVADAPAPLGEPMDLMTALQLVLR | S2 | At2g32060  At1g15930 | 84.4  83.7 | UI | |  |
| 251374 | Mitochondrial ribosomal protein S28 | | | KHEET*DDELMEELR | T115 | At3g18240 | 76.2 | LVDKHAET*DDELLEK | | (Reiland et al., 2009) |
| 717121 | Translation initiation factor 5A-3 | | | #**S***DEEHHFESKADAGASKTYPQQAGTIRK | S2 | At1g69410 | 95.0 | S*DDEHHFEASESGASK (At1g26630) | | (Reiland et al., 2009) |
| 832646 | Translation initiation factor 5A-1 | | |  | S2 | At1g13950 | 94.4 |  |  |  |
| 835953 | Translation initiation factor 5A-1 | | |  | S2 | At1g13950 | 93.7 |  |  |  |
| 724093 | Translation initiation factor 5A-1 | | | #**S***DEEQHFESKADAGASKTYPQQAGTIRK; @S*DEEQHFESKADAGASKTYPQQAGTIRK | S2 | At1g13950 | 93.8 | S*DDEHHFEASESGASK (At1g26630) | | (Reiland et al., 2009) |
| 563924 | Translational repressor Pumilio/PUF3 and related RNA-binding proteins | | | **S***GSAPPTVEGSLSSIGGLFDGTGIPGIK; SG**S***APPTVEGSLSSIGGLFDGTGIPGIK | S50 | At2g29200 | 68.7 | UI | |  |
|  |  |  |  |  | S52 |  |  | UI | |  |
| 227408 | Translational repressor Pumilio/PUF3 and related RNA-binding proteins | | |  | S29 | At2g29190 | 70.8 |  | |  |
|  |  |  |  |  | S31 |  |  |  | |  |
| 256777 | Translation elongation factor EF-1 alpha | | | **S***VEMHHEALQEALPGDNVGFNVK | S279 | At1g07920 | 96.0 | UI | |  |
| 655943 | Translation elongation factor EF-1 alpha | | |  | S279 | At1g07930 | 97.6 |  |  |  |
| 675976 | Translation elongation factor EF-1 alpha | | |  | S279 | At1g07920 | 98.2 |  |  |  |
| 655949 | Translation elongation factor EF-1 alpha | | |  | S279 | At5g60390 | 97.6 |  |  |  |
| 720367 | Translation elongation factor EF-1 alpha | | |  | S279 | At1g07940 | 97.3 |  |  |  |
| **3. Metabolism** | | | | | | | | | | |
| *Amino acid transport and metabolism (E)* | | | | | | | | | | |
| 568931 | Asparagine synthase | | | INSLPRRGS*EANWTEWESHS | S244 | At4g27450 | 91.6 | RGS*EANWSL | | (Sugiyama et al., 2008) |
|  |  |  |  | INS*LPRRGSEANWTEWESHS | S238 |  |  | VDVYNRVNS*IPR | | (Li et al., 2009) |
|  |  |  |  | AFANPPEELN**S***PASQR | S18 |  |  | UI | |  |
|  |  |  |  | TDS*EGFLCGANFK | S219 |  |  | VDS*EGVLCGANFK | | (Benschop et al., 2007) |
| 579495 | Asparagine synthase | | |  | S219 | At4g27450 | 89.2 |  |  |  |
| 658469 | Asparagine synthase | | | VDS*SGQVCGSTFKVDAETK | S216 | At3g22850 | 83.5 | VDS*SGEVCGVTFK | | (Sugiyama et al., 2008) |
|  |  |  |  | VDSS*GQVCGSTFKVDAETK | S217 |  |  | VDSS*GEVCGVTFK | | (Sugiyama et al., 2008) |
| 657667 | Asparagine synthase | | | #MLGVFSSAIVSPPDELVAAGSR**T***PSPK | T23 | At5g19140 | 89.3 | UI | |  |
|  |  |  |  | #MLGVFSSAIVSPPDELVAAG**S***RTPSPK | S21 |  |  | UI | |  |
| 648236 | Glutamate decarboxylase/sphingosine phosphate lyase | | | VLSKTAS*ESDVSVHSTFASR | S8 | At5g17330 | 92.7 | VLSHAVS*ESDVSVHSTFASR | | (Sugiyama et al., 2008) |
|  |  |  |  | VLSKTASES*DVSVHSTFASR | S10 |  |  | VLSHAVSES*DVSVHSTFASR | | (Sugiyama et al., 2008) |
| 833794 | Gamma-glutamyl phosphate reductase | | | LVNSS*FADLQKPQVDFDGK | S79 | At2g39800 | 90.4 | QLVNSS*FADLQKPQTELDGK | | (Reiland et al., 2009) |
|  |  |  |  | RLVNS*SFADLQKPQVDFDGK | S78 |  |  | QLVNS*SFADLQKPQTELDGK | | (Reiland et al., 2009) |
| 736443 | Acetylcoenzyme A carboxylase | | | VAS**S***EGEDEPTESKIPDVSSISAFMTQVSELVK | S95 | AT5G16390 | 63.1 | UI | |  |
|  |  |  |  | VA**S***SEGEDEPTESKIPDVSSISAFMTQVSELVK | S94 |  |  | UI | |  |
| *Carbohydrate transport and metabolism (G)* | | | | | | | | | | |
| 835143^(a)^ | GDP-mannose pyrophosphorylase | | |  |  |  |  |  | |  |
| 835719^(a)^ | UDP-glucose/GDP-mannose dehydrogenase | | |  |  |  |  |  | |  |
| 822067 | Phosphoglucomutase | | | ATGAFILTAS*HNPGGPNEDFGIK | S124 | At1g23190 | 93.0 | ATGAFILTAS*HNPGGPTEDFGIK | | (Sugiyama et al., 2008) |
| 832763 | Phosphoglucomutase | | |  | S124 | At1g23190 | 92.1 |  |  |  |
| 641721 | Glucose-6-phosphate 1-dehydrogenase | | | #G**S***GQWMVEKR | S3 | At5g40760 | 92.6 | UI | |  |
| 736146 | Glucose-6-phosphate 1-dehydrogenase | | |  | S3 | At5g40760 | 93.8 | UI | |  |
|  |  |  |  | SDSFSKEYE**T***VPETGCLSIIVLGASGDLAK | T25 |  |  | UI | |  |
|  |  |  |  | **S***DSFSKEYETVPETGCLSIIVLGASGDLAK | S16 |  |  | UI | |  |
| 825441 | Phosphoglycerate mutase | | | #G**S***PGQNAWKLADHPKLPK | S3 | At1g09780 | 91.1 | UI | |  |
|  |  |  |  | AHGTAVGLPSEDDMGNS*EVGHNALGAGR | S82 |  |  | AHGTAVGLPSEDDMGNS*EVGHNALGAGR | | (Carroll et al., 2008) |
| 739764 | Phosphoglycerate mutase | | |  | S82 | At1g09780 | 90.7 |  |  |  |
| 821843 | Glyceraldehyde 3-phosphate dehydrogenase | | | FGIVEGLMTTVHAITA**T***QK | T189 | At3g04120 | 95.8 | UI | |  |
|  |  |  |  | AA**S***FNIIPSSTGAAK | S208 |  |  | UI | |  |
| 575307 | Glyceraldehyde 3-phosphate dehydrogenase | | |  | S205 | At1g13440 | 95.5 |  |  |  |
| 728998 | Glyceraldehyde 3-phosphate dehydrogenase | | |  | S205 | At1g13440 | 96.1 |  |  |  |
|  |  |  |  | FGIIEGLMTTVHSITA**T***QK | T186 |  |  | UI | |  |
| 739954 | Trehalose-6-phosphate synthase component TPS1 and related subunits | | | #VSRS*YSNLLDLASGDAPIPSFGRE | S5 | At4g17770 | 90.5 | S*YSNLLDLASGNFHSFSR | | (Reiland et al., 2009) |
|  |  |  |  | SY**S***NLLDLASGDAPIPSFGR | S7 |  |  | UI | |  |
| 726767 | Trehalose-6-phosphate synthase component TPS1 and related subunits | | | SY**S***NLLELASGESPSFGR | S7 | At4g17770 | 84.4 | UI | |  |
|  |  |  |  | S*YSNLLELASGESPSFGR | S5 |  |  | S*YSNLLDLASGNFHSFSR | | (Reiland et al., 2009) |
| 656997 | Trehalose-6-phosphate synthase component TPS1 and related subunits | | | SY**S***NLLELASGESPSFER | S7 | At4g17770 | 85.0 | UI | |  |
|  |  |  |  | S*YSNLLELASGESPSFER | S5 |  |  | S*YSNLLDLASGNFHSFSR | | (Reiland et al., 2009) |
| 568670 | Trehalose-6-phosphate synthase component TPS1 and related subunits | | | VM**T***VPGVISELDDDVANSVTSDVPSSVVQDR | T34 | At1g06410 | 89.0 | UI | |  |
|  |  |  |  | S*YTNLLDLASGNFPAMGQPR | S5 |  |  | S*YTNLLDLASGNFPVMGR | | (Reiland et al., 2009) |
|  |  |  |  | SY**T***NLLDLASGNFPAMGQPR | T7 |  |  | UI | |  |
| 758739 | Trehalose-6-phosphate synthase component TPS1 and related subunits | | |  | T7 | At1g06410 | 87.8 | UI | |  |
| *Cell cycle control, cell division, chromosome partitioning (D)* | | | | | | | | | | |
| 740524^(a)^ |  | | |  |  |  |  |  | |  |
| 714870 | Protein Mei2, essential for commitment to meiosis, and related proteins | | | HFGFFPE**S***PETSFMNQVALGGMGLNR | S464 | At1g29400 | 71.6 | UI | |  |
| 410877 | Protein Mei2, essential for commitment to meiosis, and related proteins | | |  | S526 | At1g29400 | 72.0 |  |  |  |
|  |  |  |  | LFSSS*LPVLPHEK | S17 |  |  | LFSSS*LPVFPR | | (Reiland et al., 2009) |
| 204274 | SAP family cell cycle dependent phosphatase-associated protein | | | TRD**S***DEDDLHDRDYDVAALANNLSQAFR | S509 | At1g07990 | 80.7 | UI | |  |
| *Energy production and conversion (C)* | | | | | | | | | | |
| 815719 | Predicted oxidoreductase | | | A**S***PPHPNLELRPLGNTGLK | S3 | At4g33670 | 90.9 | UI | |  |
| *Inorganic ion transport and metabolism (P)* | | | | | | | | | | |
| 826518 | H+ ATPase (AHA10) **transporter** | | | NLDLNLIQTAH**T***V | T949 | At1g17260 | 90.0 | UI | |  |
| 422528 | H+ ATPase (AHA11) **transporter** | | | LKGLDIDTIQQAYT*V | T965 | At5g62670 | 96.3 | LKGLDIETIQQAYT*V | | (Benschop et al., 2007) |
| *Lipid transport and metabolism (I)* | | | | | | | | | | |
| 736443^(a)^ | 3-Methylcrotonyl-CoA carboxylase, biotin carboxylase subunit | | |  |  |  |  |  | |  |
| 287942 | phosphoglyceride transfer family protein (SEC14 proteins) | | | KVPLTLVSFKEES*NALADLSHIER | S50 | At1g72160 | 84.4 | SMIPQNLGSFKEES*SK | | (Sugiyama et al., 2008) |
| *Nucleotide transport and metabolism (F)* | | | | | | | | | | |
| 808714 | AMP deaminase | | | SHS*VSGDLHGVQPDPFAADILR | S159 | At2g38280 | 85.2 | SHSVSGDLHGVQPDPIAADILR | | (Reiland et al., 2009) |
| 560928 | AMP deaminase | | |  | S211 | At2g38280 | 84.0 |  |  |  |
| *Secondary metabolites biosynthesis, transport and catabolism (Q)* | | | | | | | | | | |
| 554850 | ABC transporter family protein^(b)^ | | | WAALEKLP**T***YDR | T55 | At1g15520 | 86.2 | UI | |  |
| 800153 | ABC transporter family protein^(b)^ | | |  | T55 | At1g15520 | 83.8 |  |  |  |
| **4. Poorly Characterized** | | | | | | | | | | |
| *Function unknown (S)* | | | | | | | | | | |
| 818850 | Dehydrin | | | KADEVPPPAPEHV**S***PEAAVSHEGDAK | S180 | At1g20440 | 59.6 | UI | |  |
| 748355 | Uncharacterized conserved protein | | | ESVQGAGQQVMS**T***AQGAVEGIK | T75 | At5g38760 | 57.6 | UI | |  |
|  |  |  |  | E**S***VVGEKTSPTMMDKAGTAAQYAK | S40 |  |  | UI | |  |
| 657068 | Uncharacterized conserved protein  (IPR_Zn-finger, C-x8-C-x5-C-x3-H type) | | | ES**S*******PGFDVLVDNELR | S258 | At2g02160 | 53.1 | UI | |  |
| 645393 | Uncharacterized conserved protein  (IPR_DNA/RNA binding protein) | | | VEKPKPES*PINENEIR | S14 | At1g76010 | 76.4 | ADT*PIDANEIR | | (Sugiyama et al., 2008) |
| 569930 | Vernalization independence 4 (VIP4) ^(b)^ | | | NLRPEDMLADEDAQYE**S***EEENR | S225 | At5g61150 | 77.1 | UI | |  |
| *General function prediction only (R)* | | | | | | | | | | |
| 743875^(a)^ |  | | |  |  |  |  |  | |  |
| 729865^(a)^ | PolyC-binding proteins alphaCP-1 and related KH domain proteins | | |  |  |  |  |  | |  |
| 662371 | Cytochrome b5 domain-containing protein^(b)^ | | | MSFEDKDLTGDVSGLGPFELEALQDWE**Y***K | Y154 | At3g48890 | 81.9 | UI | |  |
| 666994 | Cytochrome b5 domain-containing protein^(b)^ | | |  | Y154 | At3g48890 | 77.7 |  |  |  |
| 171987 | BSL3 kelch repeat-containing serine/threonine phosphoesterase family protein ^(b)^ | | | QLS*IDQFENEGR | S640 | At1g08420 | 81.4 | QLS*IDQFENEGR | | (Benschop et al., 2007) |
| 204190 | BSL3 kelch repeat-containing serine/threonine phosphoesterase family protein ^(b)^ | | |  | S517 |  | 92.8 |  |  |  |
| 588050 | Conserved Zn-finger protein | | | ESYGEEGEGHGGRSQYEKPSYGDD**S***PKR | S343 | At3g29075 | 38.3 | UI | |  |
| 577003 | Metallopeptidase | | | **S***S*DDEREERELDLTSPEVVTK | S2 | At3g51800 | 90.5 | UI | |  |
|  |  |  |  | SS*DDEREERELDLTSPEVVTK | S3 |  |  | UI | |  |
| 819223 | Metallopeptidase | | | #S***S***DDEREERELDLTSPEVITK | S2 | At3g51800 | 91.8 | UI | |  |
|  |  |  |  | #SS*DDEREERELDLTSPEVITK | S3 |  |  | UI | |  |
| 556549 | Putative bZIP transcription factor involved in embryonic development | | | SLSQPS**S***FFSLDSLPPLSPAPFR | S21 | At4g38900 | 69.3 | UI | |  |
| 553698 | FOG: RRM domain | | | #TKVGEEEIVYES*DPEEEKR | S13 | At1g80000 | 52.6 | PDGVEDS*DYES*DPDELNR | | (Reiland et al., 2009) |
| 726204 | Putative eukaryotic translation initiation factor ( eIF4B1)^(b)^ | | | SPGFSERPPS*RPGSFDESR | S464 | At1g13020 | 70.3 | GGSYSERPHS*R | | (Reiland et al., 2009) |
| 203151 | RNA-binding protein (RRM superfamily) | | | #ANTEAEAVDFEPEDDDLMDEDGAVDVDASSS*PRAPLPK | S32 | At1g51510 | 79.3 | GTAIDGADVS*PR | | (Reiland et al., 2009) |
| 172155 | RNA-binding protein (RRM superfamily) | | | #ANTEAEAVDFEPEEDDLMDEDGAADADASSS*PRAPLPK | S32 | At1g51510 | 80.7 | GTAIDGADVS*PR | | (Reiland et al., 2009) |
| **5. no KOG ID** | | | | | | | | | | |
| 765836 | None | | | TS**S***LPTETEEEWR | S95 | At1g13740 | 62.1 | UI | |  |
| 769927 | None | | |  | S95 |  | 62.8 |  |  |  |
| 656686 | None | | | AFFDS*ADWALCK | S47 | At4g16146 | 78.6 | AFFDS*ADWALLKQEASIDQR | | (Reiland et al., 2009) |
| 659041 | None | | |  | S27 |  | 72.5 |  |  |  |
| 645711 | None | | | VRSDVGAGEL**S***PSVPSTIEEGGKR | S81 | At5g64160 | 68.5 | UI | |  |
| 756582 | None | | |  | S170 |  | 49.3 |  |  |  |
| 709976 | None | | | YSIGSENSMEQ**S***PIHNHAR | S146 | At3g25070 | 53.0 | UI | |  |
| 584641 | None | | | AYFDS*ADWALGK | S56 | At5g64130 | 88.6 | AYFDS*ADWALGK | | (Reiland et al., 2009) |
| 561661 | None | | | SA**S***AKSAFSHFEEEDIVESR | S247 | At2g26110 | 60.1 | UI | |  |
| 766915 | Glycine-rich protein^(b)^ | | | #AA**T***PTSEMADGPVLSLITKR | T4 | At1g27090 | 78.0 | UI | |  |
|  |  |  |  | #AATP**T***SEMADGPVLSLITKR  #AAT*PT*SEMADGPVLSLITKR | T6 |  |  | UI | |  |
|  |  |  |  | #AATPT**S***EMADGPVLSLITKR | S7 |  |  | UI | |  |
| 652073 | Light-harvesting complex II  protein Lhcb1.2 | | | T**T***KPVPSGSPWYGPDR  T*TKPVPSGSPWYGPDR | T39 | At2g34430 | 95.5 | UI | |  |
|  |  |  |  |  | T38 |  |  | UI | |  |
| 715463 | Light-harvesting complex II  protein Lhcb1.3 | | |  | T39 | At2g34430 | 95.1 |  | |  |
|  |  |  |  |  | T38 |  |  |  | |  |
| 570481 | Protein phosphatase inhibitor 2 (IPP-2) | | | ITEPKTPYHPMIDVDDDSLS*PR | S46 | At5g52200 | 71.9 | TPYHPMMDDDGSLS*PR | | (Sugiyama et al., 2008) |
| 667000 | Protein phosphatase inhibitor 2 (IPP-2) | | |  | S43 | At5g52200 | 76.6 |  |  |  |
| 552645 | Phosphoenolpyruvate carboxylase | | | MAS*IDAQLR | S11 | At3g14940 | 93.7 | MAS*IDAQLR | | (Sugiyama et al., 2008) |
| 745223 | Phosphoenolpyruvate carboxylase | | |  | S3 |  | 95.4 |  |  |  |
| 728315 | Phosphoenolpyruvate carboxylase | | | NLEKLAS*IDAQLR | S12 | At1g53310 | 95.3 | MAS*IDVHLR | | (Reiland et al., 2009) |
| 746317 | Dihydrodipicolinate reductase family protein^(b)^ | | | MGSSVILAAN**S***AGLQILPK | S31 | At3g59890 | 91.3 | UI | |  |
| 647948 | Putative dormancy-associated protein | | | SMTMPGTPGTP**T***TPVTPTTPVSAR | T63 | At1g28330 | 85.0 | UI | |  |
|  |  |  |  | SMTMPGTPG**T***PTTPVTPTTPVSAR | T61 |  |  | UI | |  |
|  |  |  |  | SMTMPGTPGTPTTPVTPT**T***PVSAR | T70 |  |  | UI | |  |
| 568329 | Universal stress protein (Usp) | | | #AS**S***PSPKKNPPTESAVVVQVQPPS*PR | S4 | At1g11360 | 78.7 | UI | |  |
|  |  |  |  | #ASSP**S***PKKNPPTESAVVVQVQPPS*PR | S6 |  |  | UI | |  |
|  |  |  |  | KNPPTESAVVVQVQPPS*PR | S25 |  |  | SPTVVTVQPSS*PR | | (Reiland et al., 2009) |
|  |  |  |  | #ASSPSPKKNPP**T***ESAVVVQVQPPS*PR | T13 |  |  | UI | |  |
| 816463 | None | | | NTRDSG**S***GGGILSSAAAAVTNTFK | S53 | No |  | UI | |  |
|  |  |  |  | D**S***GSGGGILSSAAAAVTNTFK | S51 |  |  | UI | |  |
| 275859 | Polyphenol oxidase^(b)^ | | | FDVLVNDEPD**S***PGGPDKSEFAGSFINVPHK | S452 | No |  | UI | |  |

Underlined characters in Protein ID indicate novel phosphosites identified in these proteins. Superscript (a) in Protein ID indicates that the protein has been listed repeatedly in other subtypes; superscript (b) in the annotation indicates that the protein description was modified according to the annotation of *Arabidopsis* counterparts; the annotation of acidic ribosomal proteins was revised according to a report on poplar ribosomal proteins (Liu et al., 2010). Superscript (c) indicates that not all of the identified phosphopeptides are shown in the table because of space restrictions, * represents identified phosphorylation site, and all identified phosphopeptides are listed in Supporting information Table S1. In the phosphopeptides identified, S, T, and Y shown in bold represent novel phosphosites; C, M, #, and @ represent cysteine (C) carbamidomethylation, methionine (M) oxidation, N-terminal acetylation, and N-terminal Carbamylation, respectively. UI indicates that the phosphorylation site in poplar has not been identified it its *Arabidopsis* counterpart.
