# Supplementary material for: Identification and analysis of phosphorylation status of proteins in dormant terminal buds of poplar
Source: BMC Plant Biol. 2011 Nov 11;11:158. doi: 10.1186/1471-2229-11-158 (PMC3234192; doi:10.1186/1471-2229-11-158)
Supplement: Additional file 3 — Detailed information for phosphopeptides and phosphoproteins identified in dormant terminal buds of poplar. [file 1471-2229-11-158-S3.DOC]

## Additional file 3 - Detailed information for phosphopeptides and phosphoproteins identified in dormant terminal buds of poplar

| Protein ID | Annotation | Identified Phosphopeptides^(c)^ | Res. Pos. |
| --- | --- | --- | --- |
| **1. Cellular Processes and Signal** | | | |
| *Cell wall/membrane/envelope biogenesis (M)* | | | |
| 835143 | GDP-mannose pyrophosphorylase | RISS*FEALQSATR | S217 |
| 828302 | Glycosyl transferase family protein^(b)^ | TQ**T***AGNLGESMLDSEVVPSSLVEIAPILR | T21 |
| 578888 | Glycosyltransferase | INS*VDAMEAWVNQQK | S56 |
|  |  | EAVADMS*EDLSEGEKGDTVGDLSAHGDSVR | S133 |
|  |  | EAVADMS*EDLS*EGEKGDTVGDLSAHGDSVR | S137 |
| *Cytoskeleton (Z)* | | | |
| 263289 | Alpha tubulin | TVQFVDWCPT*GFK | T174 |
| 571195 | Tubulin alpha-6 chain |  | T349 |
| 814768 | Tubulin alpha-4 chain |  | T349 |
| 826146 | Tubulin alpha-8 chain |  | T349 |
| 831020 | Tubulin alpha-2 chain |  | T349 |
| 550223 | GAS (Growth-arrest-specific protein), and related proteins | ILAETQMASES**S***PHHYR | S589 |
| 552310 | von Willebrand factor and related coagulation proteins | AVTTNATRVP**S***PGPSLR | S350 |
|  |  | GAFMEVIR**S***PK | S215 |
| 572436 | von Willebrand factor and related coagulation proteins |  | S340 |
| *Defense mechanisms (V)* | | | |
| 552310^(a)^ |  |  |  |
| 572436^(a)^ |  |  |  |
| *Intracellular trafficking, secretion, and vesicular transport (U)* | | | |
| 573860 | Cytosolic sorting protein GGA2/TOM1 | MSAGMSSMS*FK | S35 |
| 827509 | Cytosolic sorting protein GGA2/TOM1 |  | S35 |
| 743875 | Vacuolar sorting protein VPS1, dynamin, and related proteins | HSLS*DGSLDTMAR | S714 |
| 821436 | Vesicle coat complex COPII, subunit SEC31 | LDDTPADEMGYENNQEATIFSADDGEDFFNNLP**S***PK | S523 |
| 798347 | Synaptic vesicle protein EHS-1 and related EH domain proteins | FDSFSMNEGGF**S***PR | S876 |
| 797875 | ER-Golgi vesicle-tethering protein p115 | LMELGEDVDKLLEGVGDDMGLPEDS*EKEED | S910 |
| 774347 | Rab6 GTPase-interacting protein involved in endosome-to-TGN transport | GYV**S***GDEAVIEEQK | S383 |
| *Posttranslational modification, protein turnover, chaperones (O)* | | | |
| 835143^(a)^ | GDP-mannose pyrophosphorylase |  |  |
| 657150 | Molecular chaperones HSP70/HSC70, HSP70 superfamily | LMPEPTAVALLYAQQQQQTVHENMGSG**S***EK;  LMPEPTAVALLYAQQQQQTVHENMG**S***GSEK | S224 |
|  |  |  | S222 |
| 769322 | Molecular chaperones HSP70/HSC70, HSP70 superfamily |  | S224 |
|  |  |  | S222 |
| 652330 | Molecular chaperone (HSP90 family) | EIS*DDEDEDVEDKKDEEGNVEDVDDEK | S219 |
|  |  | **T***TEKEISDDEDEDVEDKKDEEGNVEDVDDEK | T213 |
| 832078 | Molecular chaperone (small heat shock protein Hsp26/Hsp42) | IMGLDAPLFNTLQHMMDA**S***DHEADKSFNAPTR | S23 |
| 830227 | Multifunctional chaperone (14-3-3 family) | DNLTLWTSDLS*EEGGEHSTAVEPR | S237 |
| 673509 | 20S proteasome beta subunit A-1 | WHEELEPQNSLLDILSSS**S***PEPMVT | S231 |
| 819127 | 20S proteasome beta subunit A-1 | WHEELEPQNSLLDILSSS**S***PEPMVS | S231 |
| 823453 | Calnexin | EG**S***SSGDEKKEETEAENEAAAPAR | S509 |
|  |  | EGSS***S***GDEKKEETEAENEAAAPAR | S510 |
|  |  | EGS*SS*GDEKKEETEAENEAAAPAR | S511 |
| 729432 | Calreticulin | KRDEEESKEDPADS*DAEEEDEAGDAEGEDSDAETK | S379 |
| 834953 | Ubiquitin-like proteins | **S***GVTGQPQEEDKKPNDQSAHINLK | S2 |
| 711526 | Ubiquitin-like proteins | SGA**T***GQPQEEDKKPNDQSAHINLK | T5 |
|  |  | **S***GATGQPQEEDKKPNDQSAHINLK | S2 |
| 817608 | Ubiquitin-specific protease | TQ**S***FIPSELSDIFGGQLR | S104 |
| 740524 | Anaphase promoting complex, Cdc20, Cdh1, and Ama1 subunits | #SD**S***ETTPTVSTTAPPSDDHSSAEGIKK | S4 |
| 572716 | Heat shock factor binding protein | EGHDSEDPKQSTADM**T***AFVQHLLQQMQSR | T17 |
| *Signal transduction mechanisms (T)* | | | |
| 798347^(a)^ |  |  |  |
| 554898 | Protein phosphatase 2C^(b)^ | VSGMIEGLIW**S***PR | S131 |
| 587195 | Protein phosphatase 2C^(b)^ |  | S131 |
| 299214 | SNF1 kinase ^(b)^ | DGHFLKTSCGS*PNYAAPEVISGK | S179 |
| 818055 | SNF1 kinase ^(b)^ |  | S179 |
| 828986 | SNF1 kinase ^(b)^ |  | S84 |
| 422370 | Leucine-rich repeat receptor-like protein kinase (LRK)^(b)^ | VFDLEDLLRAS*AEVLGK | S336 |
| 835719 | UDP-glucose/GDP-mannose dehydrogenase | KFDWDHPLHLQPKS*PK | S393 |
| 736382 | Ca^2+^-binding protein, EF-Hand protein superfamily | SQHEKPSYGDD**S***PKRVSHEGGDYERPSYGSR | S291 |
| 570193 | Neoplastic transformation suppressor | LLDTDGESHIDRSDPNYD**S***GEEPYQLVGATISDPIDDYKK | S110 |
| **2. Information Storage and Processing** | | | |
| *RNA processing and modification (A)* | | |  |
| 414256 | Splicing factor 1/branch point binding protein (RRM superfamily) | MLQSGLPLDDRPEGAR**S***P**S***PEPIYDNMGIR | S125 |
|  |  |  | S127 |
| 423773 | Splicing factor 1/branch point binding protein (RRM superfamily) |  | S133 |
|  |  |  | S135 |
| 253138 | Splicing factor RNPS1, SR protein superfamily | VEKEMGAVQD**S***PGASATQAEKEEVDSR | S75 |
| 279975 | Splicing factor RNPS1, SR protein superfamily |  | S75 |
| 825640 | K-homology type RNA binding proteins | RQ**T***GFSSPISDPAPPPSYNSVPPPVDEIQMAK | T37 |
|  |  | RQTGFS**S***PISDPAPPPSYNSVPPPVDEIQMAK | S41 |
|  |  | RQTGF**S***SPISDPAPPPSYNSVPPPVDEIQMAK | S40 |
| 653922 | K-homology type RNA binding proteins | RQ**T***GFSSPISDPAPPPSYNSVAPPADEIQMAK | T37 |
|  |  | QTGFSSPI**S***DPAPPPSYNSVAPPADEIQMAK | S44 |
|  |  | RQTGF**S***SPISDPAPPPSYNSVAPPADEIQMAK | S40 |
|  |  | RQTGFS**S***PISDPAPPPSYNSVAPPADEIQMAK | S41 |
| 781843 | K-homology type RNA binding  proteins | KLEDLEPETLEEAEPSPADEQEPEEDGKADDVEDGG**S***PDSKR | S57 |
| 729865 | PolyC-binding proteins alphaCP-1 and related KH domain proteins | IIDGLDSDSSN**T***PPTSGAK | T81 |
| 552542 | ATP-dependent RNA helicase | AVDAGMLEYDS*DDNPVVVDKK | S157 |
| 826955 | ATP-dependent RNA helicase | EHGRGD**S***PAKSDLDGLTPFEK | S39 |
| *Transcription (K)* | | | |
| 572716^(a)^ | Heat shock factor binding protein |  |  |
| 826637 | CREB/ATF family transcription factor | SKGSLG**S***LNMITGK | S145 |
| 757220 | CREB/ATF family transcription factor |  | S145 |
| 663123^(b)^ | Dehydrin family protein | LPGGGGGMTQGG**Y***NQQEHRGGAQGGYNQQEHR | Y115 |
| 571250^(b)^ | Dehydrin family protein |  | Y115 |
| 835251 | Transcriptional coactivator p100 | RGLWVHGDIES*DDEDVLPVKK | S969 |
| 738421 | CCR4-NOT transcriptional regulation complex, NOT5 subunit | SS**S***PSLVDTGLAR | S395 |
| 717176 | Transcription factor MEIS1 and related HOX domain proteins | NQSGFSFMGSSELDGITQG**S***PKKPR | S351 |
| 563417 | Transcriptional regulators binding to the GC-rich sequences | AAAPDYISLDSGSNHQGGF**S***DEEPEFR | S259 |
| 649767 | Calcium-responsive transcription coactivator | LFLFPVNP**S***PASFGSDGGR | S149 |
| 247052 | CCAAT-binding factor, subunit A (HAP3) | #AD**S***DNES*GGHNAVSELSAKEQDRFLPIANVSR | S4 |
|  |  | ADS*DNE**S***GGHNAVSELSAK | S8 |
| *Translation, ribosomal structure and biogenesis (J)* | | | |
| 832583 | 60S acidic ribosomal protein P0A^(b)^ | KEEPAEES*DDDMGFSLFD | S312 |
| 822404 | 60S acidic ribosomal protein P0B^(b)^ |  | S310 |
| 552351 | 60S acidic ribosomal protein P1D^(b)^ | KKEEVKEES*EDEDMGFSLFD | S99 |
| 832971 | 60S acidic ribosomal protein P2B^(b)^ | KEEKVEEKEES*DDDMGFSLFD | S103 |
| 836661 | 60S acidic ribosomal protein P2C^(b)^ |  | S104 |
| 723244 | 60S acidic ribosomal protein P3A^(b)^ | KKEEEPES*DDDMGFSLFD | S106 |
| 714910 | 40S ribosomal protein S12 | **S***GEEGAVPQNETPAVADAPAPLGEPMDLMTALQLVLR | S2 |
| 251374 | Mitochondrial ribosomal protein S28 | KHEET*DDELMEELR | T115 |
| 717121 | Translation initiation factor 5A-3 | #**S***DEEHHFESKADAGASKTYPQQAGTIRK | S2 |
| 832646 | Translation initiation factor 5A-1 |  | S2 |
| 835953 | Translation initiation factor 5A-1 |  | S2 |
| 724093 | Translation initiation factor 5A-1 | #**S***DEEQHFESKADAGASKTYPQQAGTIRK; @S*DEEQHFESKADAGASKTYPQQAGTIRK | S2 |
| 563924 | Translational repressor Pumilio/PUF3 and related RNA-binding proteins | **S***GSAPPTVEGSLSSIGGLFDGTGIPGIK; SG**S***APPTVEGSLSSIGGLFDGTGIPGIK | S50 |
|  |  |  | S52 |
| 227408 | Translational repressor Pumilio/PUF3 and related RNA-binding proteins |  | S29 |
|  |  |  | S31 |
| 256777 | Translation elongation factor EF-1 alpha | **S***VEMHHEALQEALPGDNVGFNVK | S279 |
| 655943 | Translation elongation factor EF-1 alpha |  | S279 |
| 675976 | Translation elongation factor EF-1 alpha |  | S279 |
| 655949 | Translation elongation factor EF-1 alpha |  | S279 |
| 720367 | Translation elongation factor EF-1 alpha |  | S279 |
| **3. Metabolism** | | | |
| *Amino acid transport and metabolism (E)* | | | |
| 568931 | Asparagine synthase | INSLPRRGS*EANWTEWESHS | S244 |
|  |  | INS*LPRRGSEANWTEWESHS | S238 |
|  |  | AFANPPEELN**S***PASQR | S18 |
|  |  | TDS*EGFLCGANFK | S219 |
| 579495 | Asparagine synthase |  | S219 |
| 658469 | Asparagine synthase | VDS*SGQVCGSTFKVDAETK | S216 |
|  |  | VDSS*GQVCGSTFKVDAETK | S217 |
| 657667 | Asparagine synthase | #MLGVFSSAIVSPPDELVAAGSR**T***PSPK | T23 |
|  |  | #MLGVFSSAIVSPPDELVAAG**S***RTPSPK | S21 |
| 648236 | Glutamate decarboxylase/sphingosine phosphate lyase | VLSKTAS*ESDVSVHSTFASR | S8 |
|  |  | VLSKTASES*DVSVHSTFASR | S10 |
| 833794 | Gamma-glutamyl phosphate reductase | LVNSS*FADLQKPQVDFDGK | S79 |
|  |  | RLVNS*SFADLQKPQVDFDGK | S78 |
| 736443 | 3-Methylcrotonyl-CoA carboxylase, biotin carboxylase subunit | VAS**S***EGEDEPTESKIPDVSSISAFMTQVSELVK | S95 |
|  |  | VA**S***SEGEDEPTESKIPDVSSISAFMTQVSELVK | S94 |
| *Carbohydrate transport and metabolism (G)* | | | |
| 835143^(a)^ | GDP-mannose pyrophosphorylase |  |  |
| 835719^(a)^ | UDP-glucose/GDP-mannose dehydrogenase |  |  |
| 822067 | Phosphoglucomutase | ATGAFILTAS*HNPGGPNEDFGIK | S124 |
| 832763 | Phosphoglucomutase |  | S124 |
| 641721 | Glucose-6-phosphate 1-dehydrogenase | #G**S***GQWMVEKR | S3 |
| 736146 | Glucose-6-phosphate 1-dehydrogenase |  | S3 |
|  |  | SDSFSKEYE**T***VPETGCLSIIVLGASGDLAK | T25 |
|  |  | **S***DSFSKEYETVPETGCLSIIVLGASGDLAK | S16 |
| 825441 | Phosphoglycerate mutase | #G**S***PGQNAWKLADHPKLPK | S3 |
|  |  | AHGTAVGLPSEDDMGNS*EVGHNALGAGR | S82 |
| 739764 | Phosphoglycerate mutase |  | S82 |
| 821843 | Glyceraldehyde 3-phosphate dehydrogenase | FGIVEGLMTTVHAITA**T***QK | T189 |
|  |  | AA**S***FNIIPSSTGAAK | S208 |
| 575307 | Glyceraldehyde 3-phosphate dehydrogenase |  | S205 |
| 728998 | Glyceraldehyde 3-phosphate dehydrogenase |  | S205 |
|  |  | FGIIEGLMTTVHSITA**T***QK | T186 |
| 739954 | Trehalose-6-phosphate synthase component TPS1 and related subunits | #VSRS*YSNLLDLASGDAPIPSFGRE | S5 |
|  |  | SY**S***NLLDLASGDAPIPSFGR | S7 |
| 726767 | Trehalose-6-phosphate synthase component TPS1 and related subunits | SY**S***NLLELASGESPSFGR | S7 |
|  |  | S*YSNLLELASGESPSFGR | S5 |
| 656997 | Trehalose-6-phosphate synthase component TPS1 and related subunits | SY**S***NLLELASGESPSFER | S7 |
|  |  | S*YSNLLELASGESPSFER | S5 |
| 568670 | Trehalose-6-phosphate synthase component TPS1 and related subunits | VM**T***VPGVISELDDDVANSVTSDVPSSVVQDR | T34 |
|  |  | S*YTNLLDLASGNFPAMGQPR | S5 |
|  |  | SY**T***NLLDLASGNFPAMGQPR | T7 |
| 758739 | Trehalose-6-phosphate synthase component TPS1 and related subunits |  | T7 |
| *Cell cycle control, cell division, chromosome partitioning (D)* | | | |
| 740524^(a)^ |  |  |  |
| 714870 | Protein Mei2, essential for commitment to meiosis, and related proteins | HFGFFPE**S***PETSFMNQVALGGMGLNR | S464 |
| 410877 | Protein Mei2, essential for commitment to meiosis, and related proteins |  | S526 |
|  |  | LFSSS*LPVLPHEK | S17 |
| 204274 | SAP family cell cycle dependent phosphatase-associated protein | TRD**S***DEDDLHDRDYDVAALANNLSQAFR | S509 |
| *Energy production and conversion (C)* | | | |
| 815719 | Predicted oxidoreductase | A**S***PPHPNLELRPLGNTGLK | S3 |
| *Inorganic ion transport and metabolism (P)* | | | |
| 826518 | H+ ATPase (AHA10) **transporter** | NLDLNLIQTAH**T***V | T949 |
| 422528 | H+ ATPase (AHA11) **transporter** | LKGLDIDTIQQAYT*V | T965 |
| *Lipid transport and metabolism (I)* | | | |
| 736443^(a)^ | 3-Methylcrotonyl-CoA carboxylase, biotin carboxylase subunit |  |  |
| 287942 | Phosphoglyceride transfer family protein (SEC14 proteins) | KVPLTLVSFKEES*NALADLSHIER | S50 |
| *Nucleotide transport and metabolism (F)* | | | |
| 808714 | AMP deaminase | SHS*VSGDLHGVQPDPFAADILR | S159 |
| 560928 | AMP deaminase |  | S211 |
| *Secondary metabolites biosynthesis, transport and catabolism (Q)* | | | |
| 554850 | ABC transporter family protein^(b)^ | WAALEKLP**T***YDR | T55 |
| 800153 | ABC transporter family protein^(b)^ |  | T55 |
| **4. Poorly Characterized** | | | |
| *Function unknown (S)* | | | |
| 818850^(b)^ | Dehydrin family protein | KADEVPPPAPEHV**S***PEAAVSHEGDAK | S180 |
| 748355 | Uncharacterized conserved protein | ESVQGAGQQVMS**T***AQGAVEGIK | T75 |
|  |  | E**S***VVGEKTSPTMMDKAGTAAQYAK | S40 |
| 657068 | Uncharacterized conserved protein  (IPR_Zn-finger, C-x8-C-x5-C-x3-H type) | ES**S*******PGFDVLVDNELR | S258 |
| 645393 | Uncharacterized conserved protein  (IPR_DNA/RNA binding protein) | VEKPKPES*PINENEIR | S14 |
| 569930 | Vernalization independence 4 (VIP4) ^(b)^ | NLRPEDMLADEDAQYE**S***EEENR | S225 |
| *General function prediction only (R)* | | | |
| 743875^(a)^ |  |  |  |
| 729865^(a)^ | PolyC-binding proteins alphaCP-1 and related KH domain proteins |  |  |
| 662371 | Cytochrome b5 domain-containing protein^(b)^ | MSFEDKDLTGDVSGLGPFELEALQDWE**Y***K | Y154 |
| 666994 | Cytochrome b5 domain-containing protein^(b)^ |  | Y154 |
| 171987 | BSL3 kelch repeat-containing serine/threonine phosphoesterase family protein ^(b)^ | QLS*IDQFENEGR | S640 |
| 204190 | BSL3 kelch repeat-containing serine/threonine phosphoesterase family protein ^(b)^ |  | S517 |
| 588050 | Conserved Zn-finger protein | ESYGEEGEGHGGRSQYEKPSYGDD**S***PKR | S343 |
| 577003 | Metallopeptidase | **S***S*DDEREERELDLTSPEVVTK | S2 |
|  |  | SS*DDEREERELDLTSPEVVTK | S3 |
| 819223 | Metallopeptidase | #S***S***DDEREERELDLTSPEVITK | S2 |
|  |  | #SS*DDEREERELDLTSPEVITK | S3 |
| 556549 | Putative bZIP transcription factor involved in embryonic development | SLSQPS**S***FFSLDSLPPLSPAPFR | S21 |
| 553698 | FOG: RRM domain | #TKVGEEEIVYES*DPEEEKR | S13 |
| 726204 | Putative eukaryotic translation initiation factor ( eIF4B1)^(b)^ | SPGFSERPPS*RPGSFDESR | S464 |
| 203151 | RNA-binding protein (RRM superfamily) | #ANTEAEAVDFEPEDDDLMDEDGAVDVDASSS*PRAPLPK | S32 |
| 172155 | RNA-binding protein (RRM superfamily) | #ANTEAEAVDFEPEEDDLMDEDGAADADASSS*PRAPLPK | S32 |
| **5. no KOG ID** | |  |  |
| 765836 | None | TS**S***LPTETEEEWR | S95 |
| 769927 | None |  | S95 |
| 656686 | None | AFFDS*ADWALCK | S47 |
| 659041 | None |  | S27 |
| 645711 | None | VRSDVGAGEL**S***PSVPSTIEEGGKR | S81 |
| 756582 | None |  | S170 |
| 709976 | None | YSIGSENSMEQ**S***PIHNHAR | S146 |
| 584641 | None | AYFDS*ADWALGK | S56 |
| 561661 | None | SA**S***AKSAFSHFEEEDIVESR | S247 |
| 766915 | Glycine-rich protein^(b)^ | #AA**T***PTSEMADGPVLSLITKR | T4 |
|  |  | #AATP**T***SEMADGPVLSLITKR  #AAT*PT*SEMADGPVLSLITKR | T6 |
|  |  | #AATPT**S***EMADGPVLSLITKR | S7 |
| 652073 | Light-harvesting complex II  protein Lhcb1.2 | T**T***KPVPSGSPWYGPDR  T*TKPVPSGSPWYGPDR | T39 |
|  |  |  | T38 |
| 715463 | Light-harvesting complex II  protein Lhcb1.3 |  | T39 |
|  |  |  | T38 |
| 570481 | Protein phosphatase inhibitor 2 (IPP-2) | ITEPKTPYHPMIDVDDDSLS*PR | S46 |
| 667000 | Protein phosphatase inhibitor 2 (IPP-2) |  | S43 |
| 552645 | Phosphoenolpyruvate carboxylase | MAS*IDAQLR | S11 |
| 745223 | Phosphoenolpyruvate carboxylase |  | S3 |
| 728315 | Phosphoenolpyruvate carboxylase | NLEKLAS*IDAQLR | S12 |
| 746317 | Dihydrodipicolinate reductase family protein^(b)^ | MGSSVILAAN**S***AGLQILPK | S31 |
| 647948 | Putative dormancy-associated protein | SMTMPGTPGTP**T***TPVTPTTPVSAR | T63 |
|  |  | SMTMPGTPG**T***PTTPVTPTTPVSAR | T61 |
|  |  | SMTMPGTPGTPTTPVTPT**T***PVSAR | T70 |
| 568329 | Universal stress protein (Usp) | #AS**S***PSPKKNPPTESAVVVQVQPPS*PR | S4 |
|  |  | #ASSP**S***PKKNPPTESAVVVQVQPPS*PR | S6 |
|  |  | KNPPTESAVVVQVQPPS*PR | S25 |
|  |  | #ASSPSPKKNPP**T***ESAVVVQVQPPS*PR | T13 |
| 816463 | None | NTRDSG**S***GGGILSSAAAAVTNTFK | S53 |
|  |  | D**S***GSGGGILSSAAAAVTNTFK | S51 |
| 275859 | Polyphenol oxidase^(b)^ | FDVLVNDEPD**S***PGGPDKSEFAGSFINVPHK | S452 |

Underlined characters in Protein ID indicate novel phosphoproteins identified in these proteins. Superscript (a) in Protein ID indicates that the protein has been listed repeatedly in other subtypes; superscript (b) in the annotation indicates that the protein description was modified according to the annotation of *Arabidopsis* counterparts; the annotation of acidic ribosomal proteins was revised according to a report on poplar ribosomal proteins (Liu et al., 2010). Superscript (c) indicates that not all identified phosphopeptides are listed in the table because of space restrictions, * represents identified phosphorylation site, and all identified phosphopeptides are listed in Supporting information Table S1. In the phosphopeptides identified, S, T, and Y shown in bold represent novel phosphosites; C, M, #, and @ represent cysteine (C) carbamidomethylation, methionine (M) oxidation, N-terminal acetylation, and N-terminal carbamylation, respectively. UI indicates that the phosphorylation site in poplar protein has not been identified in its *Arabidopsis* counterpart.
