# Supplementary material for: Identification and analysis of phosphorylation status of proteins in dormant terminal buds of poplar
Source: BMC Plant Biol. 2011 Nov 11;11:158. doi: 10.1186/1471-2229-11-158 (PMC3234192; doi:10.1186/1471-2229-11-158)
Supplement: Additional file 5 — Comparison of singly and doubly phosphorylated peptides. [file 1471-2229-11-158-S5.DOC]

| **Additional file 5.** Comparison of singly and doubly phosphorylated peptides | | | | | | | |
| --- | --- | --- | --- | --- | --- | --- | --- |
| All | | pS | | pT | | pY | |
| Single | Double | Single | Double | Single | Double | Single | Double |
| 151 (93.8%) | 10 (6.2%) | 122 (93.1%) | 9 (6.9%) | 27 (93.1%) | 2 (6.9%) | 2 (100%) | 0 (0%) |
