# Supplementary material for: Identification and analysis of phosphorylation status of proteins in dormant terminal buds of poplar
Source: BMC Plant Biol. 2011 Nov 11;11:158. doi: 10.1186/1471-2229-11-158 (PMC3234192; doi:10.1186/1471-2229-11-158)
Supplement: Additional file 6 — Location of phosphorylation sites in characterized conserved domains. [file 1471-2229-11-158-S6.DOC]

| **Additional file 6.** Location of phosphorylation sites on characterized conserved domains | | | | |
| --- | --- | --- | --- | --- |
| Number of proteins with Pfam domain | | Number of phosphorylation sites | | |
|  |  | Pfam domain^a^ | | Total (%) |
|  |  | ON (%) | OUT^b^ (%) |  |
| pS | 114 | 49 (16.1) | 255 (83.9) | 304 (100) |
| pT | 25 | 12 (23.1) | 40 (76.9) | 52 (100) |
| pY | 4 | 4 (100) | 0 (0.0) | 4 (100) |
| All | 134 | 65 (18.1) | 295 (81.9) | 360 (100) |
| ^a^We analyzed whether phosphorylation sites were located on conserved domains annotated in the Pfam database.  ^b^A single phosphorylation site can be counted more than once, because several domains can be present in a single phosphoprotein. | | | | |
