# Supplementary figures and images for: Identification and analysis of phosphorylation status of proteins in dormant terminal buds of poplar
Source: BMC Plant Biol. 2011 Nov 11;11:158. doi: 10.1186/1471-2229-11-158 (PMC3234192; doi:10.1186/1471-2229-11-158)

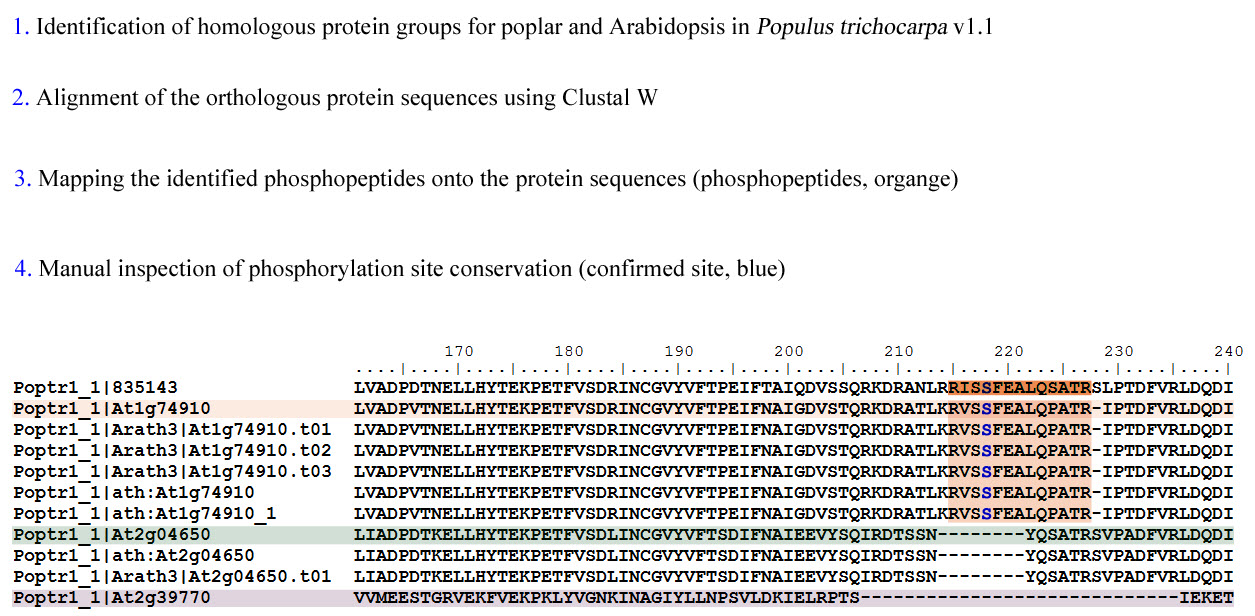

Supplement: Additional file 7 — Flowchart for analyzing the conservation of phosphoproteins and phosphosites between poplar and Arabidopsis. [file 1471-2229-11-158-S7.JPEG]

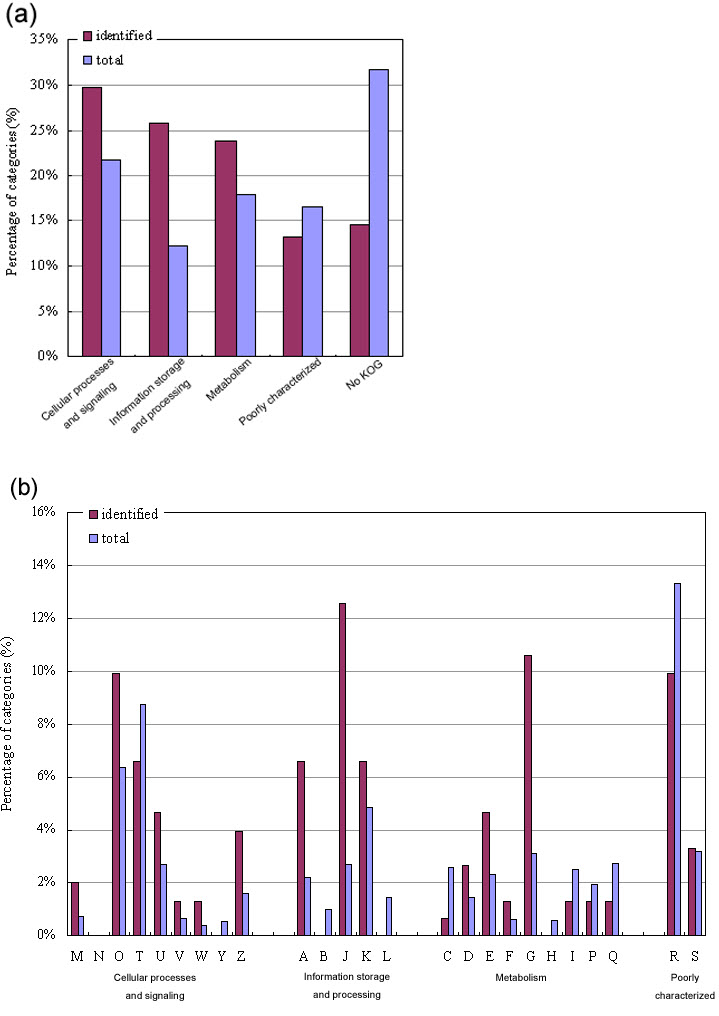

Supplement: Additional file 10 — KOG analysis of identified phosphoproteins and all proteins encoded in Populus trichocarpa genome. (a) Percentage of KOG functional group categories from the identified phosphoproteins and all proteins encoded in Populus trichocarpa genome. (b) Percentage of KOG functional subgroup categories from the identified phosphoproteins and all proteins encoded in Populus trichocarpa genome. [file 1471-2229-11-158-S10.JPEG]

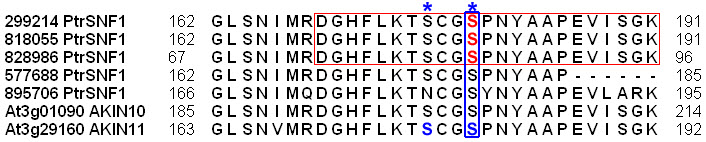

Supplement: Additional file 12 — Sequence alignment of phosphorylated sites in protein kinases between poplar and Arabidopsis. [file 1471-2229-11-158-S12.JPEG]

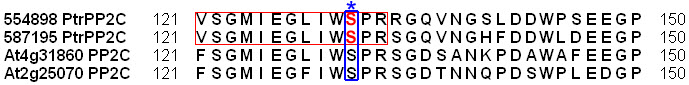

Supplement: Additional file 14 — Sequence alignment of phosphorylated sites in protein phosphatases between poplar and Arabidopsis. [file 1471-2229-11-158-S14.JPEG]

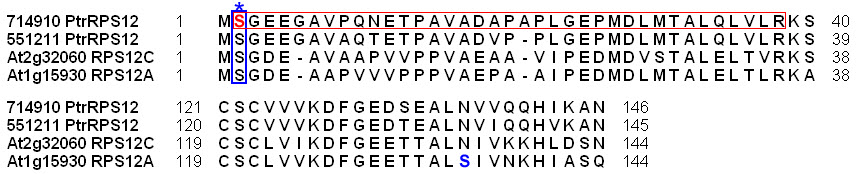

Supplement: Additional file 15 — Sequence alignment of RPS12 between poplar and Arabidopsis. [file 1471-2229-11-158-S15.JPEG]

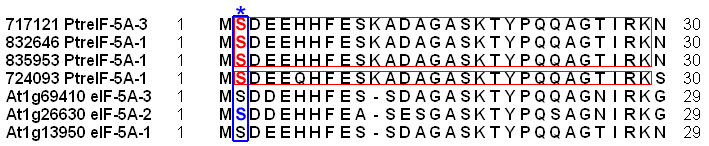

Supplement: Additional file 16 — Sequence alignment of conserved N-terminus of eIF5A between poplar and Arabidopsis. [file 1471-2229-11-158-S16.JPEG]

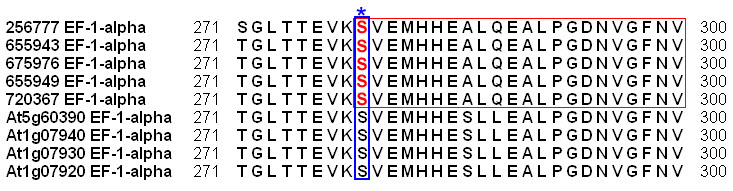

Supplement: Additional file 17 — Sequence alignment of conserved C-terminus of EF-1-alpha between poplar and Arabidopsis. [file 1471-2229-11-158-S17.JPEG]
