# Supplementary material for: Identification and analysis of phosphorylation status of proteins in dormant terminal buds of poplar
Source: BMC Plant Biol. 2011 Nov 11;11:158. doi: 10.1186/1471-2229-11-158 (PMC3234192; doi:10.1186/1471-2229-11-158)
Supplement: Additional file 8 — Conserved phosphorylation sites within orthologous proteins. (a) Phosphosites conserved in orthologous proteins. (b) Phosphosites that were not conserved in orthologous proteins. [file 1471-2229-11-158-S8.DOC]

| **Additional file 8.** Conserved phosphorylation sites within orthologous proteins | | |
| --- | --- | --- |
| Poplar protein No. | Poplar (JGI ID)  Arabidopsis (AGI ID) | Alignment of identified phosphopeptides in our study |
| **(a). Phosphosites identified in orthologous proteins** | | |
| 1 | 835143  At1g74910.t02 | RIS**S**FEALQSATR  RVS**S**FEALQPATR |
| 3 | 578888  At5g20280.t01 | EAVADM**S**EDL**S**EGEKGDTVGDLSAHGDSVR  EATADM**S**EEF**S**EGEKGDIISDISTHGESTK |
|  | 578888  At5g20280.t01 | IN**S**VDAMEAWVNQQK  IN**S**AESMELWASQQK |
| 4,5,6,7,8 | 263289  571195  814768  826146  831020  At5g19770  At5g19780.t01 | TVQFVDWCP**T**GFK  TVQFVDWCP**T**GFK  TVQFVDWCP**T**GFK  TVQFVDWCP**T**GFK  TVQFVDWCP**T**GFK  TVQFVDWCP**T**GFK  TVQFVDWCP**T**GFK |
| 12,13 | 573860  827509  At5g16880.t03 | MSAGMSSM**S**FK  MSAGMSSM**S**FK  ISAGVSSM**S**FK |
| 14 | 743875  At1g59610.t01 | HSL**S**DGSLDTMAR  QSL**S**EGSLDKMVR |
| 17 | 797875  At3g27530.t01 | LMELGEDVDKLLEGVGDDMGLPED**S**EKE  LIELGVDVDKLLEDIGDESEAQAE**S**EED |
| 21 | 652330  At5g56030.t01 | EI**S**DDEDEDVEDKKDEEGNVEDVDDEK  EI**S**DDEEE--EEKKDEEGKVEEVDEEK |
| 26 | 823453  At5g61790.t01 | EG**SSS**GDEKKEETEAENEAAAPAR  --SK**S**GD----EAEKKEETAAPRK |
| 27 | 729432  At1g56340.t01 | KRDEEESKEDPADSDAEEEDEAGDAEGED**S**DAETK  KREEEESKDAPAESDAEEEAEDDDNEGDD**S**DNESK |
| 33,34 | 554898  587195  At4g31860.t02  At2g25070 | VSGMIEGLIW**S**PR  VSGMIEGLIW**S**PR  FSGMIEGLIW**S**PR  FSGMIEGFIW**S**PR |
| 35,36,37 | 299214  818055  828986  At3g29160.t02 | DGHFLKTSCG**S**PNYAAPEVISGK  DGHFLKTSCG**S**PNYAAPEVISGK  DGHFLKTSCG**S**PNYAAPEVISGK  DGHFLKTSCG**S**PNYAAPEVISGK |
| 38 | 422370  At1g48480 | VFDLEDLLRA**S**AEVLGK  VFDLEDLLRA**S**AEVLGK |
| 39 | 835719  At3g29360 | KFDWDHPLHLQPK**S**P  KFDWDHPLHLQPM**S**P |
| 50 | 552542  At2g47330.t01 | AVDAGMLEYD**S**DDNPVVVDKK  AVDAGMLDYD**S**DDNPIVVDKR |
| 56 | 835251  At5g07350.t01 | GLWVHGDIE**S**DDEDVLPVKK  GIWQYGDIE**S**DDEDTGPARK |
| 62,63 | 832583  822404  At3g09200 | KEEPAEE**S**DDDMGFSLFD  KEEPAEE**S**DDDMGFSLFD  VEEKEE**S**DEEDYGGDFGLFDEE |
| 64 | 552351  At1g01100 | KKEEVKEE**S**EDEDMGFSLFD  KKDEPAEE**S**DGDLGFGLFD |
| 65,66 | 832971  836661  At2g27720 | KEEKVEEKEE**S**DDDMGFSLFD  KEEKVEEKEE**S**DDDMGFSLFD  EE**S**DDDMGFSLFE |
| 67 | 723244  At4g25890 | KKEEEPE**S**DDDMGFSLFD  KKEE**S**EEEEGDFGFDLFG |
| 69 | 251374  At3g18240.t02 | KHEE**T**DDELMEELR  KHAE**T**DDELLEKIE |
| 81,82 | 568931  579495  At4g27450 | TD**S**EGFLCGANFK  TD**S**EGFLCGANFK  VD**S**EGVLCGANFK |
|  | 568931  At4g27450 | IN**S**LPRRG**S**EANWT  VN**S**IPRRG**S**EANWS |
| 83 | 658469  At3g22850.t01 | VD**SS**GQVCGSTFKVDAETK  VD**SS**GEVCGVTFKVDSEAK |
| 85 | 648236  At5g17330 | VLSKTA**S**E**S**DVSVHSTFASR  VLSHAV**S**E**S**DVSVHSTFASR |
| 86 | 833794  At2g39800.t02 | RLVN**SS**FADLQKPQVDFDGK  QLVN**SS**FADLQKPQTELDGK |
| 88,89 | 822067  832763  At1g23190 | ATGAFILTA**S**HNPGGPNEDFGIK  ATGAFILTA**S**HNPGGPNEDFGIK  ATGAFILTA**S**HNPGGPTEDFGIK |
| 92,93 | 825441  739764  At1g09780 | AHGTAVGLP**S**EDDMGNSEVGHNALGAGR  AHGTAVGLP**S**EDDMGNSEVGHNALGAGR  AHGTAVGLP**S**EDDMGNSEVGHNALGAGR |
| 102,103 | 410877  At1g29400 | LFSS**S**LPVLPHEK  LFSS**S**LPVFPRGK |
| 106,107 | 826518  422528  At5g62670 | NLDLNLIQTAH**T**V  GLDIDTIQQAY**T**V  GLDIETIQQAY**T**V |
| 108 | 287942  At1g72160 | KVPLTLVSFKEE**S**NALADLSHIER  MIPQNLGSFKEE**S**SKLSDLSNSEK |
| 116 | 645393  At1g76010 | VEKPKPE**S**PINENEIR  VVKPKAD**T**PIDANEIR |
| 120,121 | 171987  204190  At1g08420 | QL**S**IDQFENEGR  QL**S**IDQFENEGR  QL**S**IDQFENEGR |
| 126 | 553698  At1g80000.t02 | TKVGEEEIVYE**S**DPEEEKR  APDGVED**S**DYE**S**DPDELNR |
| 127 | 726204  At1g13020.t01 | SPGFSERPP**S**RPGSFDESR  GGSYSERPH**S**RAGSIDESR |
| 128,129 | 203151  172155  At1g51510 | ANTEAEAVDFEPEDDDLMDEDGAVDVDASS**S**PRAPLPK  ANTEAEAVDFEPEEDDLMDEDGAADADASS**S**PRAPLPK  ANIESEAVDFEPEEDDLMDEEGTAIDGADV**S**PRAGHPR |
| 132,133,137 | 656686  659041  584641  At4g16146  At5g64130.t01 | AFFD**S**ADWALCK  AFFD**S**ADWALCK  AYFD**S**ADWALGK  AFFD**S**ADWALLK  AYFD**S**ADWALGK |
| 140,141 | 652073  715463  At2g34430 | **TT**KPV-PSGSPWYGPDR  **TT**KPV-PSGSPWYGPDR  A**S**KPTGPSGSPWYGSDR |
| 142,143 | 570481  667000  At5g52200.t01 | TPYHPMIDVDDDSL**S**PR  TPYHPMIDVDDDSL**S**PR  PYHPMM-D-DDGSL**S**PR |
| 144,145,146 | 552645  745223  728315  At3g14940.t01  At1g53310.t01 | MA**S**IDAQLR  MA**S**IDAQLR  LA**S**IDAQLR  MA**S**IDAQLR  MA**S**IDVHLR |
| 149 | 568329  At1g11360.t01 | S**S**P**S**PKKNPP**T**ESAVVVQVQPP**S**PR  TSPGKSPRSDRKSPTVVTVQPS**S**PR |
| **(b). Phosphosites that have not been identified in orthologous proteins** | | |
| 2 | 828302  At2g31960.t01 | TQ**T**AGNLGESMLDSEVVPSSLVEIAPILR  TQ**T**AGNLGEAMLDSEVVPSSLVEIAPILR |
| 9 | 550223  At5g55860.t01 | ILAETQ--MASES**S**P-HHYR  ILAEAEMKMASES**S**PQQHYK |
| 16 | 798347  At1g20760.t01 | FDSFSMNEGG---F**S**PR  FDSFNTSEAGAGFS**S**QP |
| 19,20 | 657150  769322  At2g32120.t02 | LMPEPTAVALLYAQQQQQTVHENMG**S**G**S**EK  LMPEPTAVALLYAQQQQQTVHENMG**S**G**S**EK  LMPEPTAIALLYAQQQQMTTHDNMG**S**G**S**ER |
| 21 | 652330  At5g56030.t01 | **T**TEKEISDDEDEDVEDKKDEEGNVEDVDDEK  **T**IEKEISDDEEE--EEKKDEEGKVEEVDEEK |
| 26 | 823453  At5g61790.t01 | EG**S**SSGDEKKEETEAENEAAAPAR  --**S**KSGD----EAEKKEETAAPRK |
| 28,29 | 834953  711526  At4g26840.t01 | **S**GVTGQPQEEDKKPNDQSAHINLK  **S**GATGQPQEEDKKPNDQSAHINLK  **S**AN----QEEDKKPGDGGAHINLK |
| 30 | 817608  At4g30890.t02 | TQ**S**FIPSELSDIFGGQLR  TQ**S**FVPSELSEIFGGQLK |
| 32 | 572716  At4g15802 | EGHDSEDPKQSTADM**T**AFVQHLLQQMQSR  DGHDSEDTKQSTADM**T**AFVQNLLQQMQTR |
| 41 | 570193  At5g63190.t01 | LLDT-DGESHIDRSDPNYD**S**GEEPYQ-LVGATISDPIDDYKK  LLDTDDGDSCIDKNDPNYD**S**GEDAYDGLVDSPVSDPLNDYKK |
| 42,43 | 414256  423773  At5g51300.t01 | MLQSGLPLDDRPEGAR**S**P**S**PEPIYDNMGIR  MLQSGLPLDDRPEGAR**S**P**S**PEPIYDNMGIR  MLQSGMPLDDRPEGQR**S**P**S**PEPVYDNMGIR |
| 44,45 | 253138  279975  At5g51120 | VEKEMGAVQD-**S**PGASATQAEKEEVDSR  VEKEMGAVQD-**S**PGASATQAEKEEVDSR  AEKDMGASQDP**S**GGVSA--AEKEEVDSR |
| 46,47 | 825640  653922  At2g25970.t01 | RQ**T**GF**SS**-PI-S---DPA-----PPPSYNSVPPPVD  RQ**T**GF**SS**-PI-**S**---DPA-----PPPSYNSVAPPAD  RP**T**GF**SS**GPIP**S**ASVDPTAPTGLPPSSYNSVPPPMD |
| 48 | 781843  At4g10070.t01 | KLEDLEPETLEEAEPSPADEQEPEEDGKADDVEDGG**S**PDSKR  KL----------------DEVELNGNIVDDSNQTSD**S**SQAKR |
| 51 | 826955  At1g55150 | REHGRGD**S**PAKSDLDGLTPFEK  KDNDGNE**S**PRKLDLDGLTPFEK |
| 52,53 | 826637  757220  At2g35530 | SKGSLG**S**LNMITGK  SKGSLG**S**LNMITGK  SRGSLG**S**LNMITGK |
| 58 | 717176  At2g35940.t01 | NQSGFSFMGSSELDGIT--QG**S**PKKPR  ----------GDLEGVTGMQG**S**PKRLR |
| 59(49.2%) | 563417  At5g08550.t01 | AA-APDYISLDSG-SNHQG—-GF**S**DEEPEFR  SAPAPDYISLDGGIVNHSAVEGV**S**DEDADFQ |
| 60 | 649767  At5g64430.t01 | LFLFPVNP**S**PASFGSDGG-R  LFLFPAS-**S**--GFGSQSSTQ |
| 61 | 247052  At4g14540 | MAD**S**DNE**S**GGHNAVSELSAK  MAD**S**DND**S**GGHKDGGNASTR |
| 68 | 714910  At2g32060.t01 | **S**GEEGAVPQNETPAVADAPAPLGEPMDLMTALQLVLR  **S**GDE-AVAAPVVPPVAEA-AVIPEDMDVSTALELTVR |
| 70,71,72,73 | 717121  832646  835953  724093  At1g69410  At1g13950.t01 | M**S**DEEHHFESKADAGASKTYPQQAGTIRK  M**S**DEEHHFESKADAGASKTYPQQAGTIRK  M**S**DEEHHFESKADAGASKTYPQQAGTIRK  M**S**DEEQHFESKADAGASKTYPQQAGTIRK  M**S**DDEHHFES-SDAGASKTYPQQAGNIRK  M**S**DEEHHFES-SDAGASKTYPQQAGTIRK |
| 74,75 | 563924  227408  At2g29200.t01  At2g29190 | **S**G**S**APPTVEGSLSSIGGLFDGTGIP-----GIK  **S**G**S**APPTVEGSLSSIGGLFDGTGIP-----GIK  **S**G**S**APPTVDGSVSAAGGLFSGGGGAPFLEFGGV  **S**G**S**APPTVDGSVSAAGGLFSGGGGAPFLEFGGG |
| 76,77,78,79,80 | 256777  655943  675976  655949  720367  At1g07920  At1g07930.t01  At5g60390 | **S**VEMHHEALQEALPGDNVGFNVK  **S**VEMHHEALQEALPGDNVGFNVK  **S**VEMHHEALQEALPGDNVGFNVK  **S**VEMHHEALQEALPGDNVGFNVK  **S**VEMHHEALQEALPGDNVGFNVK  **S**VEMHHESLLEALPGDNVGFNVK  **S**VEMHHESLLEALPGDNVGFNVK  **S**VEMHHESLLEALPGDNVGFNVK |
| 81,82 | 568931  At4g27450 | AFANPPEELN**S**PASQR  AFAHPPEELN**S**PASEK |
| 84 | 657667  At5g19140 | MLGVFSSAIVSPPDELVAAG**S**R**T**PSPK  MLGIFSGAIVSPPEELVAAG**S**R**T**PSPK |
| 90,91 | 641721  736146  At5g40760.t01 | G**S**GQWMVEKR  G**S**GQWMVEKR  G**S**GQWHVEKR |
| 92,93 | 825441  At1g09780 | G-**S**PGQNAWKLADHPKLPK  AT**S**---SAWKLDDHPKLPK |
| 94,95,96 | 821843  728998  At3g04120.t01 | FGIVEGLMTTVHAITA**T**QK  FGIIEGLMTTVHSITA**T**QK  FGIVEGLMTTVHSITA**T**QK |
|  | 821843  575307  728998  At3g04120.t01  At1g13440.t01 | AA**S**FNIIPSSTGAAK  AA**S**FNIIPSSTGAAK  AA**S**FNIIPSSTGAAK  AA**S**FNIIPSSTGAAK  AA**S**FNIIPSSTGAAK |
| 97,98,99,100,101 | 739954  726767  656997  568670  758739  At4g17770.t01  At1g06410.t01 | SY**S**NLLDLASGDAPIPSFGRE  SY**S**NLLELASGESPSFGRM  SY**S**NLLELASGESPSFER  SY**T**NLLDLASGNFPAMGQPR  SY**T**NLLDLASGNFPAMGQPR  SY**S**NLLDLASGNFHSFSR  SY**T**NLLDLASGNFPVMG--R |
|  | 568670  At1g06410.t01 | VM**T**VPGVISELDDDVANSVTSDVPSSVVQDR  VM**T**VPGNVSEFDEDQAYSVSSDNPSSVSSDR |
| 102,103 | 714870  410877  At1g29400 | HFGFFPE**S**PETS-FMNQVALGGM-GLNR  HFGFFPE**S**PETS-FMNQVALGGM-GLNR  HFGFVPE**S**SKDALFMNTVGLQGMSGMGL |
| 104 | 204274  At1g07990 | TRD**S**DEDDLHDRDYDVAALANNLSQAFR  TRD**S**DEE---DRDYDVAALANNLSQAFR |
| 109,110 | 808714  560928  At2g38280.t02 | SH**S**VSGDLHGVQPDPFAADILR  SH**S**VSGDLHGVQPDPFAADILR  **S**H**S**VSGDLHGVQPDPIAADILR |
| 111,112 | 554850  800153  At1g15520.t01 | WAALEKLP**T**YDR  WAALEKLP**T**YDR  WAALEKLP**T**FDR |
| 115 | 657068  At2g02160.t01 | ES**S**PGFDVLVDNELR  ES**S**PGFDVLVDNEAG |
| 117 | 569930  At5g61150.t01 | NLRPEDMLAD-----EDAQYE**S**EEENR  DLRPDDMVLDDIIPEEDPQYE**S**EAEHV |
| 118,119 | 662371  666994  At3g48890.t01 | MSFEDKDLTGDVSGLGPFELEALQDWE**Y**K  MSFEDKDLTGDVSGLGPFELEALQDWE**Y**K  MSFEDQDLTGDISGLGAFELEALQDWE**Y**K |
| 123,124 | 577003  819223  At3g51800.t01 | **SS**DDEREERELDLTSPEVVTKYK  **SS**DDEREERELDLTSPEVITKYK  **SS**DDERDEKELSL**TS**PEVVTKYK |
| 125 | 556549  At4g38900.t02 | SLSQPS**S**FFSLDSLPPLSPAPFR  SMSQPN**S**FFSFDSLPPLSPSPFR |
| 130,131 | 765836  769927  At1g13740.t01 | TS**S**LPTETEEEWR  TS**S**LPTETEEEWR  TT**S**LPAEMEEEWR |
| 138 | 561661  At2g26110.t01 | SA**S**AKSAFSHFEEEDI-VESR  **S**A**S**TKSPFSHFQEDEISVEAR |
| 139 | 766915  At1g27090.t01 | AA**T**P**TS**EMADGPVLSLITKR  AA**T**A**SS**EASEGPVMGLINKR |
| 147 | 746317  At3g59890.t01 | MGSSVILAAN**S**AGLQILPK  MGKAVIKAAD**S**AGVNIVPT |
| 148 | 647948  At1g28330.t02 | SMTMPG--TPG**T**P**T**TPVTPT**T**PVSAR  SLTMPAAVSPG**T**P**T**TPTTPT**T**P--RK |
| 149 | 568329  At1g11360.t01 | S**S**PSPKKNPPTESAVVVQVQPPSPR  T**S**PGKSPRSDRKSPTVVTVQPSSPR |
