# Supplementary material for: Identification and analysis of phosphorylation status of proteins in dormant terminal buds of poplar
Source: BMC Plant Biol. 2011 Nov 11;11:158. doi: 10.1186/1471-2229-11-158 (PMC3234192; doi:10.1186/1471-2229-11-158)
Supplement: Additional file 9 — Unconserved phosphorylation sites within orthologous proteins. [file 1471-2229-11-158-S9.DOC]

| **Additional file 9.** Unconserved phosphorylation sites in orthologous proteins | | |
| --- | --- | --- |
| Poplar protein No. | Poplar (JGI ID)  Arabidopsis (AGI ID) | Alignment of identified phosphopeptides in our study |
| 15 | 821436  At3g63460.t02 | LDDTPADEMGYENNQEATIFSADDGEDFFNNLP**S**-PK  LEDTAADALDLDDSNEAAAFAMDNGEDFFNNFPAKPD |
| 18 | 774347  At4g00640.t01 | GYV**S**GDEAVIEEQK  -------------- |
| 22 | 832078  At5g12020.t01 | PLFNTLQHMMDA-**S**DHEADKSFNAPTR  PIISILEDMLEVPEDHNNEKTRNNPSR |
| 23 | 830227  At1g22300.t02 | DNLTLWTSDL**S**EEGGEHST-AVEPR  DNLTLWT**S**DLNEEGDERTKGADEPQ |
| 24,25 | 673509  819127  At4g31300.t01 | WHEELEPQNSLLDILSSS**S**PEPM  WHEELEPQNSLLDILSSS**S**PEPM  WHEELEPQNSLLDILNAAGPEPM |
| 26 | 823453  At5g61790.t01 | EGS**S**SGDEKKEETEAENEAAAPAR  --SKSGD----EAEKKEETAAPRK |
| 28,29 | 834953  711526  At4g26840.t01 | SGVTGQPQEEDKKPNDQSAHINLK  SGA**T**GQPQEEDKKPNDQSAHINLK  SAN----QEEDKKPGDGGAHINLK |
| 31 | 740524  At1g47200.t01 | **S**ETTPTVSTTAPPSDDHSSAEGIKK  AETINTTISSPPPESESSTTISAMT |
| 49 | 729865  At3g04610.t01 | IIDGLDSDSSN**T**PPTSGAK  IVDGLDGEASQAPPPS--K |
| 57 | 738421  At5g18230.t01 | SS**S**PSLVDTGLAR  KPPSSVADTPL-R |
| 87 | 736443  At5g15530 | VA**S**SEGEDEPTESKIPDVSSISAFMTQVSELVK  MTNGGYMNGKAKTNVPEPAELSEFMAKVSGLLK |
| 91 | 736146  At5g40760.t01 | **S**DSFSKEYE**T**VPETGCLSIIVLGASGDLAK  ND**S**FVREYGIVPETGCLSIIVLGASGDLAK |
| 105 | 815719  At4g33670.t01 | A**S**PPHPNLELRPLGNTGLK  TK-----IELRALGNTGLK |
| 113 | 818850  At1g20440.t01 | KADE------VPPPAPEHV**S**PEAAVSHEGDAK  KAEDSPAVTSTPLVVTEHPVEPTTELPVEHPE |
| 114 | 748355  At5g38760.t01 | E**S**VVGEKTSPTMMDKAGTAAQYA  ----------TMMDKASNAAQSA |
|  | 748355  At5g38760.t01 | ESVQGAGQQVMS**T**AQGAVEGIK  ESLKETGQQIKEKAQGATESVK |
| 134,135 | 645711  756582  At5g64160.t01 | VRSDVGAGEL**S**PSVPSTIEEGGKR  VRSDVGAGEL**S**PSVPSTIEEGGKR  VRNDIDA-------PPSSESGEKR |
| 136 | 709976  At3g25070 | YSIGSENSMEQ**S**PIHNHAR  ------------------- |
| 140,141 | 652073  715463  At2g34430 | **T**TKPV-PSGSPWYGPDR  **T**TKPV-PSGSPWYGPDR  ASKPTGPSGSPWYGSDR |
| 149 | 568329  At1g11360.t01 | SSP**S**PKKNPP**T**ESAVVVQVQPPSPR  TSPGKSPRSDRKSPTVVTVQPSSPR |
