# Supplementary material for: Identification and analysis of phosphorylation status of proteins in dormant terminal buds of poplar
Source: BMC Plant Biol. 2011 Nov 11;11:158. doi: 10.1186/1471-2229-11-158 (PMC3234192; doi:10.1186/1471-2229-11-158)
Supplement: Additional file 11 — Complete list of KOG analysis of phosphoproteins and all proteins encoded in Populus trichocarpa genome. [file 1471-2229-11-158-S11.DOC]

| **Additional file 11.** A complete list of KOG analysis of the phosphoproteins and all proteins encoded by the *Populus trichocarpa* genome | | | | | |
| --- | --- | --- | --- | --- | --- |
|  |  | total | | identified | |
|  | Abbr. | Numbers | Percentage | Numbers | Percentage |
| **1. Cellular processes and signaling** |  | **9884** | **21.7%** | **45** | **29.8%** |
| Cell wall/membrane/envelope biogenesis | M | 330 | 0.7% | 3 | 2.0% |
| Cell motility | N | 7 | 0.0% | 0 | 0.0% |
| Posttranslational modification, protein turnover, chaperones | O | 2887 | 6.3% | 15 | 9.9% |
| Signal transduction mechanisms | T | 3980 | 8.7% | 10 | 6.6% |
| Intracellular trafficking, secretion, and vesicular transport | U | 1220 | 2.7% | 7 | 4.6% |
| Defense mechanisms | V | 295 | 0.6% | 2 | 1.3% |
| Extracellular structures | W | 183 | 0.4% | 2 | 1.3% |
| Nuclear structure | Y | 240 | 0.5% | 0 | 0.0% |
| Cytoskeleton | Z | 742 | 1.6% | 6 | 4.0% |
| **2. Information storage and processing** |  | **5545** | **12.2%** | **39** | **25.8%** |
| RNA processing and modification | A | 1006 | 2.2% | 10 | 6.6% |
| Chromatin structure and dynamics | B | 453 | 1.0% | 0 | 0.0% |
| Translation, ribosomal structure and biogenesis | J | 1228 | 2.7% | 19 | 12.6% |
| Transcription | K | 2214 | 4.9% | 10 | 6.6% |
| Replication, recombination and repair | L | 644 | 1.4% | 0 | 0.0% |
| **3. Metabolism** |  | **8130** | **17.8%** | **36** | **23.8%** |
| Energy production and conversion | C | 1170 | 2.6% | 1 | 0.7% |
| Cell cycle control, cell division, chromosome partitioning | D | 659 | 1.4% | 4 | 2.6% |
| Amino acid transport and metabolism | E | 1049 | 2.3% | 7 | 4.6% |
| Nucleotide transport and metabolism | F | 287 | 0.6% | 2 | 1.3% |
| Carbohydrate transport and metabolism | G | 1421 | 3.1% | 16 | 10.6% |
| Coenzyme transport and metabolism | H | 261 | 0.6% | 0 | 0.0% |
| Lipid transport and metabolism | I | 1148 | 2.5% | 2 | 1.3% |
| Inorganic ion transport and metabolism | P | 889 | 2.0% | 2 | 1.3% |
| Secondary metabolites biosynthesis, transport and catabolism | Q | 1246 | 2.7% | 2 | 1.3% |
| **4. Poorly characterized** |  | **7521** | **16.5%** | **20** | **13.2%** |
| General function prediction only | R | 6078 | 13.3% | 15 | 9.9% |
| Function unknown | S | 1443 | 3.2% | 5 | 3.3% |
| **5. No KOG** |  | **14475** | **31.8%** | **22** | **14.6%** |
