# Supplementary material for: Identification and analysis of phosphorylation status of proteins in dormant terminal buds of poplar
Source: BMC Plant Biol. 2011 Nov 11;11:158. doi: 10.1186/1471-2229-11-158 (PMC3234192; doi:10.1186/1471-2229-11-158)
Supplement: Additional file 13 — Detailed information for identified phosphoproteins referred to in discussion section. [file 1471-2229-11-158-S13.DOC]

## Additional file 13 - Detailed information for identified phosphoproteins referred to in the Discussion section

| JGI  Protein ID | NCBI  Protein ID | Best hit in TAIR  (Similarity %) | Annotation | Biological processes |
| --- | --- | --- | --- | --- |
| 299214 | XP_002337631.1 | AT3G29160.1 (95) | SNF1 kinase | Signal transduction |
| 818055 | XP_002306053.1 | AT3G01090.3 (91) | SNF1 kinase | Signal transduction |
| 828986 | XP_002334067.1 | AT3G01090.3 (91) | SNF1 kinase | Signal transduction |
| 422370 | XP_002317861.1 | AT1G48480.1 (80) | RKL1, receptor-like kinase 1 | Signal transduction |
| 554898 | XP_002304734.1 | AT4G31860.1 (84) | Protein phosphatase 2C family protein | Signal transduction |
| 587195 | XP_002332050.1 | AT2G25070.1 (85) | Protein phosphatase 2C family protein | Signal transduction |
| 729432 | XP_002318957.1 | AT1G56340.1 (89) | Calreticulin 1a | Signal transduction for cadmium ion |
| 823453 | XP_002318189.1 | [AT5G61790](http://www.arabidopsis.org/servlets/TairObject?type=locus&name=AT5G61790).1 (86) | Calnexin 1 (CNX1) | Signal transduction for cadmium ion |
| 647948 | XP_002305123.1 | AT1G28330.1 (85) | Auxin-repressed/dormancy-associated protein | Response to auxin |
| 569930 | XP_002318586.1 | AT5G61150.2 (77) | Vernalization independence 4 (VIP4) | Regulation of flower development |
| 714870 | XP_002306739.1 | AT1G29400.2 (72) | ML5, MEI2-like protein 5 | Regulation of growth and meiosis |
| 410877 | XP_002302143.1 | AT1G29400.2 (72) | ML5, MEI2-like protein 5 | Regulation of growth and meiosis |
| 736443 | XP_002323896.1 | AT5G16390.1 (63) | Acetylcoenzyme A carboxylase | Flavanoid biosynthesis |
| 275859 | XP_002331714.1 | NA | Polyphenol oxidase | Flavanoid biosynthesis |
| 818850 | XP_002307768.1 | AT1G20440.1 (60) | Dehydrin family protein | Response to cold stress |
| 663123 | XP_002319714.1 | AT1G54410.1 (57) | Dehydrin family protein | Response to cold stress |
| 571250 | XP_002319713.1 | AT1G54410.1 (57) | Dehydrin family protein | Response to cold stress |
| 657150 | XP_002311619.1 | [AT2G32120](http://www.arabidopsis.org/servlets/TairObject?type=locus&name=AT2G32120).2 (88) | Heat-shock protein 70 | Response to cold stress |
| 769322 | XP_002315776.1 | [AT2G32120](http://www.arabidopsis.org/servlets/TairObject?type=locus&name=AT2G32120).2 (87) | Heat-shock protein 70 | Response to cold stress |
| 652330 | XP_002307846.1 | AT5G56030.1 (96) | Heat-shock protein 90 | Response to cold stress |
| 832078 | XP_002308537.1 | [AT5G12020](http://www.arabidopsis.org/servlets/TairObject?type=locus&name=AT5G12020).1 (84) | Small heat-shock protein Hsp26 | Response to cold stress |
| 826518 | XP_002326625.1 | AT1G17260.1 (90) | H+ ATPase (AHA10) transporter | Transport |
| 422528 | XP_002318614.1 | AT5G62670.1 (96) | H+ ATPase (AHA11) transporter | Transport |
| 554850 | XP_002304713.1 | AT1G15520.1 (85) | ABC transporter family protein | Transport |
| 800153 | XP_002304711.1 | AT1G15520.1 (83) | ABC transporter family protein | Transport |
| 832583 | XP_002312149.1 | AT3G09200.1 (92) | 60S acidic ribosomal protein P0A | Protein synthesis |
| 822404 | XP_002315165.1 | AT3G09200.1 (92) | 60S acidic ribosomal protein P0B | Protein synthesis |
| 552351 | XP_002301441.1 | AT1G01100.4 (77) | 60S acidic ribosomal protein P1D | Protein synthesis |
| 832971 | XP_002313663.1 | AT2G27720.1 (82) | 60S acidic ribosomal protein P2B | Protein synthesis |
| 836661 | XP_002328062.1 | AT2G27720.1 (80) | 60S acidic ribosomal protein P2C | Protein synthesis |
| 723244 | XP_002314174.1 | AT4G25890.1 (77) | 60S acidic ribosomal protein P3A | Protein synthesis |
| 714910 | XP_002306757.1 | AT2G32060.2 (84) | 40S ribosomal protein S12 | Protein synthesis |
| 717121 | XP_002308400.1 | AT1G69410.1 (94) | Translation initiation factor 5A-3 | Protein synthesis |
| 832646 | XP_002312268.1 | AT1G13950.1 (94) | Translation initiation factor 5A-1 | Protein synthesis |
| 835953 | XP_002325136.1 | AT1G13950.1 (93) | Translation initiation factor 5A-1 | Protein synthesis |
| 724093 | XP_002315023.1 | AT1G13950.1 (93) | Translation initiation factor 5A-1 | Protein synthesis |
| 256777 | XP_002528028.1 | AT1G07920.1 (96) | Translation elongation factor 1A | Protein synthesis |
| 655943 | XP_002311106.1 | AT5G60390.3 (97) | Translation elongation factor 1A | Protein synthesis |
| 675976 | XP_002326338.1 | AT1G07920.1 (98) | Translation elongation factor 1A | Protein synthesis |
| 655949 | XP_002311107.1 | AT5G60390.3 (98) | Translation elongation factor 1A | Protein synthesis |
| 720367 | XP_002311107.1 | AT1G07920.1 (97) | Translation elongation factor 1A | Protein synthesis |
| 821843 | XP_002315632.1 | AT3G04120.1 (96) | GAPC, glyceraldehyde-3-phosphate dehydrogenase | Electron transport or energy pathways |
| 575307 | XP_002321679.1 | AT1G13440.1 (96) | GAPC, glyceraldehyde-3-phosphate dehydrogenase | Electron transport or energy pathways |
| 728998 | XP_002318114.1 | AT1G13440.1 (96) | GAPC, glyceraldehyde-3-phosphate dehydrogenase | Electron transport or energy pathways |
| 552645 | XP_002302820.1 | AT3G14940.1 (94) | PEPC1, phosphoenolpyruvate carboxylase 1 | Electron transport or energy pathways |
| 745223 | XP_002330719.1 | AT3G14940.1 (95) | PEPC3, phosphoenolpyruvate carboxylase 3 | Electron transport or energy pathways |
| 728315 | XP_002316861.1 | AT1G53310.3 (95) | PEPC1, phosphoenolpyruvate carboxylase 1 | Electron transport or energy pathways |
| 652073 | XP_002306927.1 | AT2G34430.1 (95) | Light-harvesting complex II protein Lhcb1.2 | Electron transport or energy pathways |
| 715463 | XP_002307725.1 | AT2G34430.1 (95) | Light-harvesting complex II protein Lhcb1.3 | Electron transport or energy pathways |

NA indicates that the poplar phosphoprotein does not have a counterpart in *Arabidopsis*.
